# Mitochondrial dysfunction activates lysosomal-dependent mitophagy selectively in cancer cells

**DOI:** 10.18632/oncotarget.23171

**Published:** 2017-12-11

**Authors:** Thomas G. Biel, V. Ashutosh Rao

**Affiliations:** ^1^ Laboratory of Applied Biochemistry, Division of Biotechnology Review and Research III, Office of Biotechnology Products, Center for Drug Evaluation and Research, Silver Spring, MD 20993, USA

**Keywords:** autophagy, mitophagy, mitochondria, mitoquinone, cancer

## Abstract

Molecules designed to target and accumulate in the mitochondria are an emerging therapeutic approach for cancer and other indications. Mitochondria-targeted redox agents (MTAs) induce mitochondrial damage and autophagy in cancer cells. However, the mechanisms for these molecules to induce mitophagy, the clearance of damaged mitochondria, are largely unknown. Using breast derived cell lines and a series of targeted molecules, mitochondrial dysfunction and autophagy was established to be selective for MDA-MB-231 cancer cells as compared to the non-cancerous MCF-12A cells. Kinetic analyses revealed that mitochondrial dysfunction precedes the activation of autophagy in these cancer cells. To determine the onset of mitophagy, stably expressing mitochondrial mKeima, a mitochondrial pH sensor, cell lines were generated and revealed that these drugs activate lysosomal dependent mitochondrial degradation in MDA-MB-231 cells. Mitophagy was confirmed by identifying the accumulation of a PINK1, mitochondria located in autophagosomes, and the formation of an autophagosome-mitochondria protein (MFN2-LC3-II) complex. These results are the first to demonstrate that mitochondrial redox agents selectively induce mitophagy in a breast cancer cell line and their potential application both as tools for investigating mitochondrial biomechanics and as therapeutic strategies that target mitochondrial metabolism.

## INTRODUCTION

Mitochondria-targeted redox molecules utilizing triphenylphosphonium (TPP) conjugation are an emerging class of experimental therapeutics to ameliorate cardiovascular disease, neurodegenerative disorder and cancer [1–5]. TPP, a lipophilic cation, facilitates mitochondrial inner membrane localization to enhance therapeutic efficiency in a manner that is dependent on mitochondrial membrane potential (ΔΨ_m_) [3–8]. As cancer cells possess hyperpolarized ΔΨ_m_, TPP conjugates selectively accumulate in the mitochondria of cancer cells by a 10 fold increase as compared to normal cells [3, 9, 10]. Mitoquinone (MitoQ), a prototype mitochondria-targeted redox agent (MTA), is a TPP conjugate that was initially designed to suppress reactive oxygen species (ROS) production and lipid peroxidation [11–13]. In contrast to the beneficial effects demonstrated in normal cells, cancer cells subjected to MitoQ underwent cell cycle arrest, mitochondrial depolarization, enhanced superoxide production and apoptosis [1, 14]. Similar to MitoQ, other TPP-conjugated drugs have also demonstrated detrimental effects that were selective for cancer cells as compared to normal cells [2, 15–18]. However, the mechanisms that contribute to the cancer cell selectivity of MTAs are not well understood.

Mitochondrial autophagy or mitophagy is the selective recognition and degradation of damaged mitochondria using the autophagic pathway [19]. Mitophagy is a critical mechanism for maintaining cellular bioenergetics and homeostasis by preventing the accumulation of free radical producing dysfunctional mitochondria [20]. In cancer, mitophagy has been demonstrated to be a pro- and an anti-tumorigenic mechanism based on the stage of the tumor [21–23]. Loss-of-function genetic mutations in *PARK2*, a crucial regulatory mitophagy gene, have been identified in several different human tumor types including triple negative breast cancer [24]. Moreover, genetic ablation of mitophagy in mice led to spontaneous hepatic tumor development, which supports mitophagy as an anti-tumorigenic mechanism [23]. In contrast to tumorigenesis, advanced glycolytic and malignant tumors have been proposed to utilize mitophagy for stress adaption and survival under hypoxic conditions to support tumor growth [21]. The mechanism that controls the mitophagy turning point from anti- to pro-tumorigenic remains unclear and how anti-cancer therapeutics designed to target or those that intentionally or unintentionally damage the mitochondria affect mitophagy in such glycolytic tumors is unknown.

Mitochondrial dysfunction is a causative factor that initiates mitophagy via PTEN inducible Kinase 1 (PINK1) signaling [25]. The loss in ΔΨ_m_ leads to the accumulation of PINK1 on the outer mitochondrial membrane [26]. PINK1 phosphorylates ubiquitin and outer mitochondrial membrane proteins to facilitate the activation and mitochondrial translocation of Parkin, an E3 ubiquitin ligase, to initiate the recognition of dysfunctional mitochondria for selective mitophagy [25, 27–29]. Using a cancerous and non-cancerous breast cell line model, the onset and mechanism(s) contributing to mitophagy in cells treated with MTAs were investigated to identify a potential target in cancer cells as compared to healthy cells. Here, we demonstrate that mitochondrial dysfunction induced by MTAs leads to activation of mitophagy selectively in a malignant breast cancer cell line.

## RESULTS

### Mitochondria-targeted redox agents induce mitochondrial depolarization and activate autophagy

**Figure 1:**
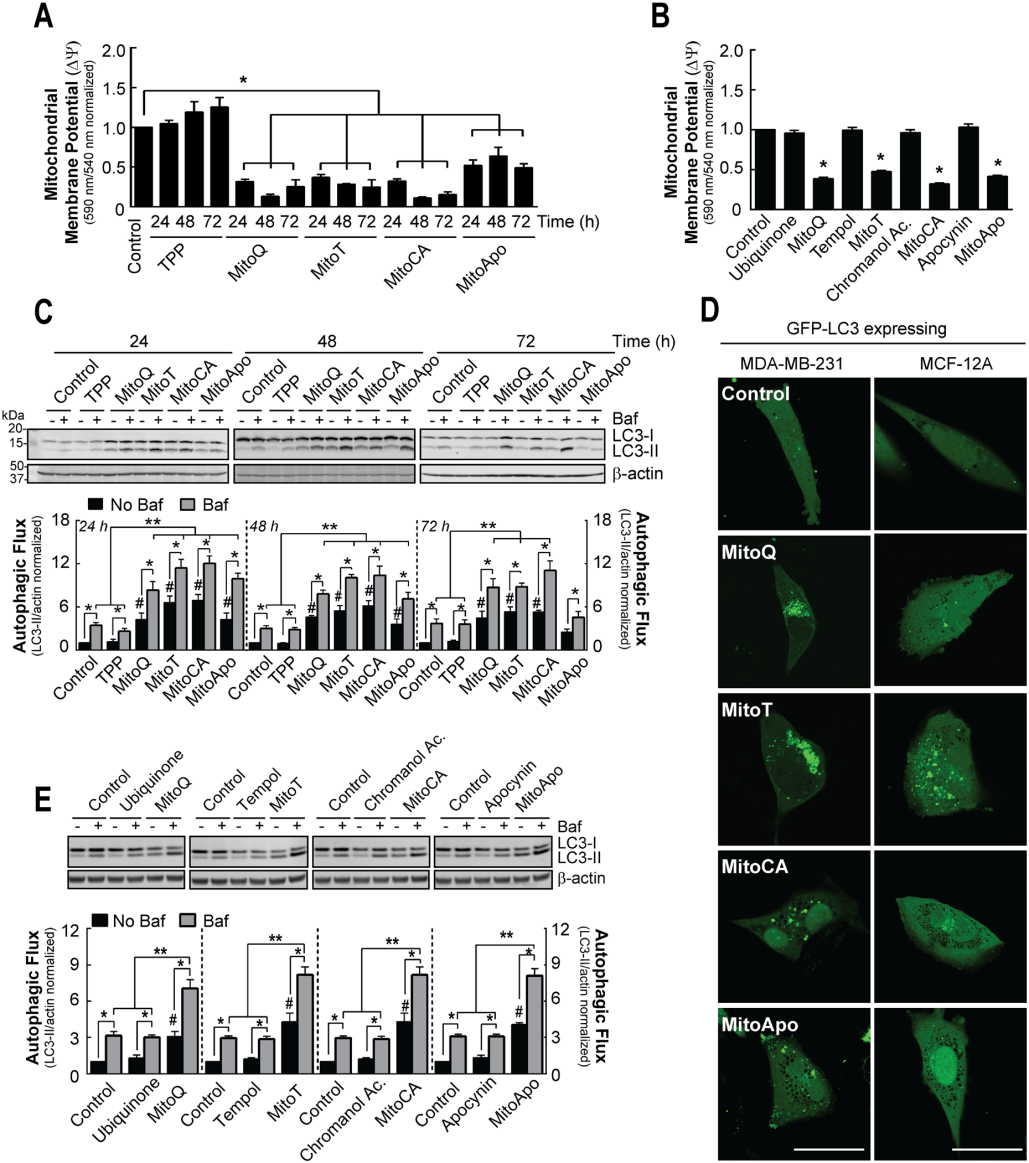
Prolonged MTA exposed MDA-MB-231 cells do not recover mitochondrial membrane potential but increase autophagic flux in MDA-MB-231 cells **(A)** MDA-MB-231 cells were subjected to DMSO (control), TPP, MitoQ, MitoT, MitoCA or MitoApo at 1 μM to analyze changes in mitochondrial membrane potential using JC-1 fluorometry at the indicated times. Bar represents mean ± SEM. (*n*=3) **(B)** Mitochondrial membrane potential was measured in MDA-MB-231 cells exposed to DMSO, ubiquinone, MitoQ, tempol, MitoT, chromanol acetate, MitoCA, apocynin or MitoApo at 1 μM for 24 hours using JC-1 fluorometry. Bars represent mean ± SEM. (*n*=3) **(C)** Representative cropped immunoblot of LC3 from MDA-MB-231 cells subjected to DMSO TPP, MitoQ, MitoT, MitoCA or MitoApo at 1 μM for 24, 48 and 72 hours with and without 5 nM Baf. Cells were treated with Baf for 2 hours prior to protein harvest. Bar represents mean ± SEM. (*n*=3-5) **(D)** Representative images of GFP-LC3 expressing MDA-MB-231 and MCF-12A cells subjected to DMSO, MitoQ, MitoT, MitoCA or MitoApo at 1 μM for 24 hours. Scale bar is 40 μM. **(E)** Representative cropped LC3 immunoblot from cells treated with redox active agents conjugated or unconjugated to the TPP for 24 hours. Cells were subjected to 5 nM Baf for 2 hours prior to protein harvest. Bar represents mean ± SEM. (*n*=3) ^*^P<0.05, and ^**^P<0.01 indicate statistical significance. ^#^*P*<0.05 indicates statistical significance between control and MTA treated cells in the absence of Baf.

To determine if different redox moieties conjugated to TPP induce mitochondrial damage and activate autophagy in breast cancer cells, the ΔΨ_m_ and autophagic flux were measured in MDA-MB-231 cells exposed to sub-lethal concentrations of MitoQ, Mitotempol (MitoT), Mitochromanol acetate (MitoCA) and Mitoapocynin (MitoApo) (Supplementary Figure 1a) [30]. Ratiometrics of JC-1 fluorescent emission shifts from 590 nm (aggregated) to 540 nm (monomer) were used to measure changes in ΔΨ_m_. Consistent with previous findings [1, 30], JC-1 aggregate formation decreased in MDA-MB-231 cells treated with MitoQ and MitoCA at 24 hours as compared to the vehicle control (Figure 1A). Similar to MitoQ and MitoCA, cells treated with MitoT, and MitoApo decreased JC-1 aggregate formation at 24 hours. Prolonging the treatments to 72 hours did not increase JC-1 monomers. TPP and unconjugated redox active agents did not affect the JC-1 fluorometric spectra (Figure 1B and Supplementary Figure 1b).

To assess for autophagy flux, cells were exposed to MTAs in the presence and absence of Bafilomycin A1 (Baf), a Vacuolar-ATPase inhibitor [31, 32], and changes in microtubule associated protein light chain 3 (LC3) were measured (Figure 1C). Cytosolic LC3-I is lipidated to form LC3-II, which is localized on autophagosomal membrane [33–36]. Subsequently, autophagosomes fuse with lysosomes leading to the degradation of LC3-II [32]. Baf-induced lysosomal neutralization results in an accumulation of LC3-II and provides a method to measure changes in autophagic flux [37]. Consistent with previous results [1], MitoQ treatment led to a 4.5 fold increase in LC3-II levels at 24 hours as compared to the vehicle control. The addition of Baf resulted in an 8.3 fold increase in LC3-II. This indicates that MitoQ does not block the fusion of autophagosomes with the lysosome. A similar response was measured for MDA-MB-231 cells treated with MitoT, MitoCA, and MitoApo as indicated by 6.6, 6.9 and 4.3 fold increases in LC3-II in the absence of Baf, and 11.4, 12.0 and 9.9 fold increases in the presence of Baf at 24 hours, respectively. At 48 hours following MTA treatment, MDA-MB-231 cells sustained an increase in autophagic flux as compared to the control. Fold increases in LC3-II levels for MitoQ, MitoT, MitoCA and MitoApo treatments at 48 hours were similar to the 24 hours. At 72 hours, MitoQ, MitoT and MitoCA treated cells retained elevated LC3-II levels and the presence of Baf led to a greater LC3-II accumulation. MitoApo was the only MTA that recovered LC3-II levels similar to the control at 72 hours. MTA induced autophagosome formation was confirmed in GFP-LC3 expressing MDA-MB-231 cells using confocal microscopy (Figure 1D). TPP alone or unconjugated redox active agents did not affect the autophagic flux in MDA-MB-231 cells (Figure 1E). Collectively, these data suggest that each MTA induced mitochondrial depolarization and autophagy in MDA-MB-231 cells.

### MTA-induced autophagy is selective for MDA-MB-231 cancer cells as compared to MCF-12A normal cells

**Figure 2:**
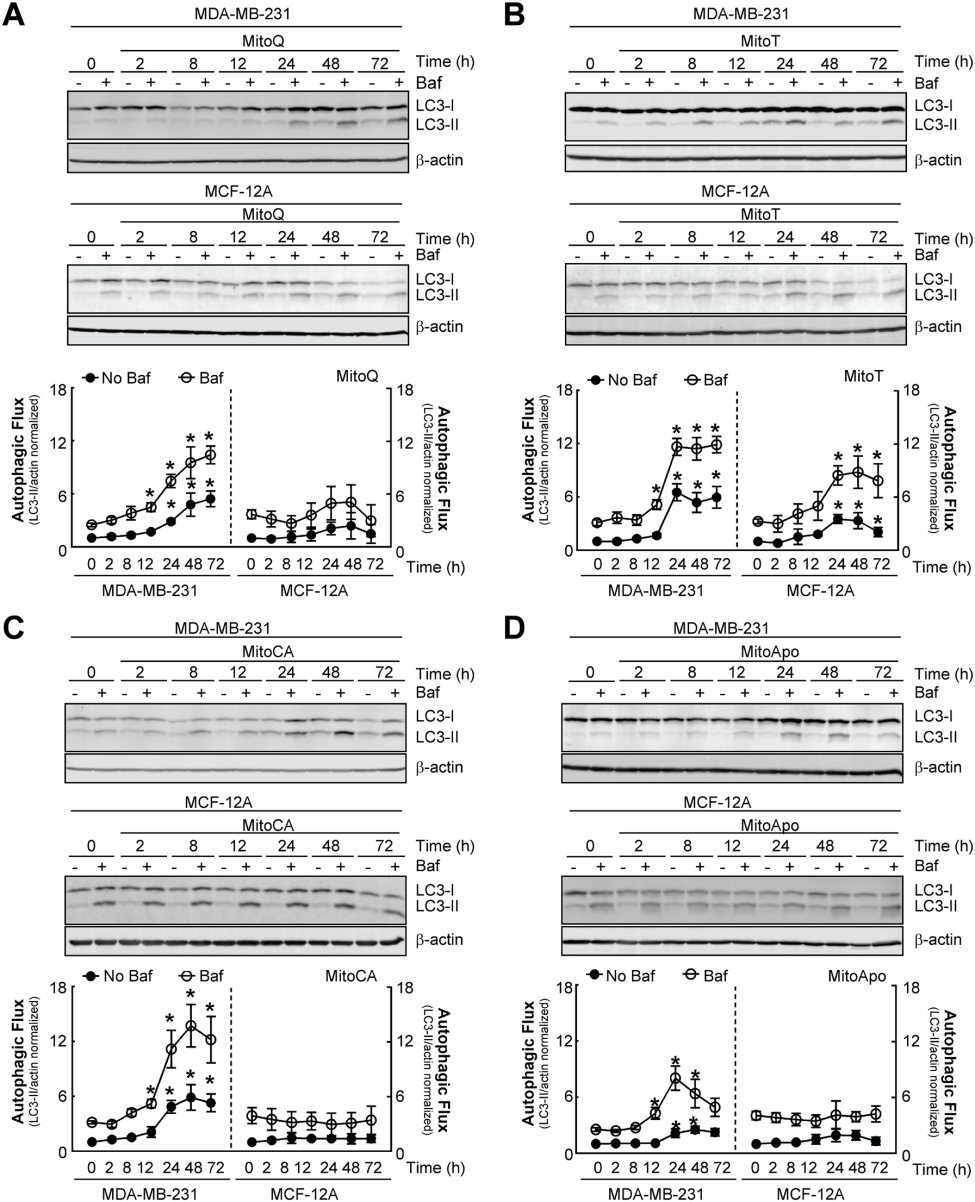
MTA-induced autophagy is selective for MDA-MB-231 cells as compared to MCF-12A cells **(A-D)** Autophagic flux kinetic analyses using LC3 immunoblotting from MDA-MB-231 and MCF-12A cells exposed to 1 μM of (A) MitoQ, (B) MitoT, (C) MitoCA or (D) MitoApo in the presence and absence of 5 nM Baf at the indicated times. Baf was added for 2 hours prior to protein harvest. Bars represent mean ± SEM. (*n*=4) ^*^P<0.05 indicates statistical significance.

MitoQ selectively inhibits cell cycle progression in MDA-MB-231 cells as compared to MCF-12A cells, a non-cancerous primary mammary cell line [1]. To determine if MTA-induced autophagy is selective for the MDA-MB-231 cells, both cell lines were treated with the MTA series to assess the autophagic flux kinetics (Figure 2). Consistent with Figure 1, each MTA increased autophagic flux in MDA-MB-231 cells at 24, and 48 hours (Figure 2A-2D). However, acute (<8 hours) MTA exposure did not enhance LC3-II accumulation in the presence of lysosomal inhibition, which required a prolonged (>12 hours) treatment. This suggests that an immediate (<8 hours) increase in autophagy does not occur in MTA treated MDA-MB-231 cells.

In contrast to the MDA-MB-231 cells, MCF-12A cells treated with MitoQ, MitoCA, and MitoApo did not increase LC3-II levels in the absence of Baf and retained a similar LC3-II accumulation in the presence of lysosomal inhibition as compared to the vehicle control (Figure 2A, 2C, and 2D). MitoT was the only MTA that caused the accumulation of LC3-II in MCF-12A cells in the absence and presence of Baf (Figure 2B). Confocal microscopy confirmed the formation of autophagosomes in GFP-LC3 expressing MCF-12A cells subjected to MitoT (Figure 1D). Furthermore, MitoT was the only MTA that caused a loss of JC-1 aggregate formation in MCF-12A cells (Supplementary Figure 2). Together, these data indicate that MitoQ, MitoCA, and MitoApo, but not MitoT, induce autophagy and mitochondrial depolarization selectively in MDA-MB-231 cells as compared to MCF-12A.

### MDA-MB-231 cells are more sensitive to MTA-induced mitochondrial damage compared to MCF-12A cells

**Figure 3:**
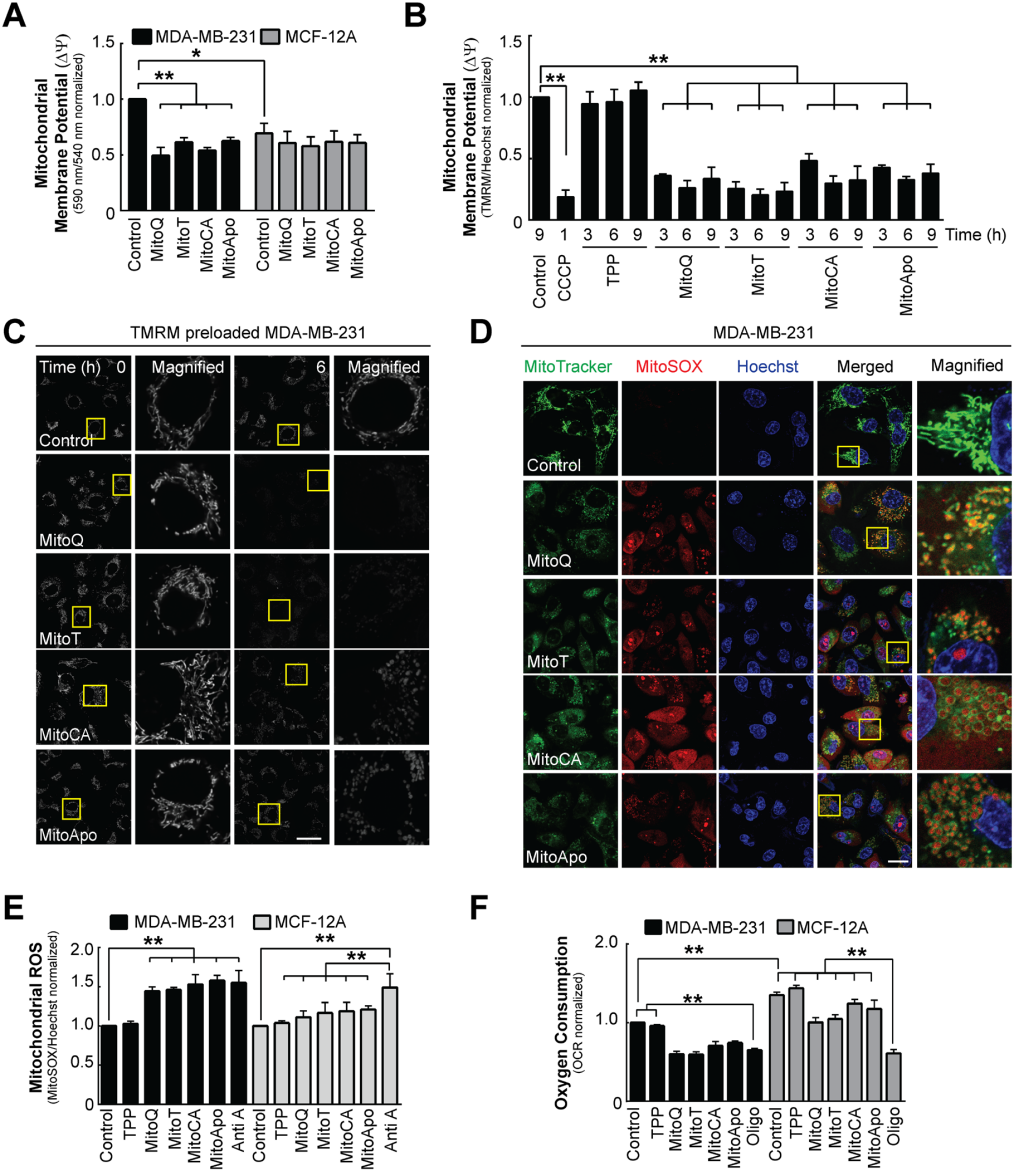
Changes in mitochondrial homeostasis as indicated by membrane depolarization, mitochondrial ROS generation and reduced oxygen consumption in MDA-MB-231 and MCF-12A cells MDA-MB-231 and MCF-12A cells were treated with DMSO (control), MitoQ, MitoT, MitoCA or MitoApo at 1 μM to evaluate changes in mitochondrial membrane potential, ROS production, morphology and function at 6 hours post treatment. **(A)** Using the JC-1 fluorometry, mitochondrial membrane potential was determined in cells treated with different MTAs. Bars represent mean ± SEM. (*n*=3) **(B)** TMRM (100 nM) preloaded MDA-MB-231 cells were exposed to different MTAs and TPP for the 3, 6 and 9 hours or CCCP (10 μM) for 1 hour to quantify the change in mitochondrial membrane potential using fluorometrics. Bars represent mean ± SEM. (*n*=3) **(C)** Representative confocal images of TMRM stained mitochondria in MDA-MB-231 cells treated with the different MTAs. Scale bar is 40 μM. Yellow box indicates area that was magnified. **(D)** Representative confocal images of MitoTracker Green (100 nM), MitoSox (2 nM) and Hoechst (5 μM) stained cells after a 6 hour treatment with different MTAs. Scale bar is 40 μM. Yellow box indicates area that was magnified. **(E)** Quantification of MitoSOX fluorescence in MDA-MB-231 and MCF-12A cells at 6 hours post MTA treatment. Bars represent mean ± SD. (*n*=3) MitoSOX emission intensity was normalized to Hoechst emission intensity. **(F)** Mitochondrial oxygen consumption in MDA-MB-231 cells subjected to different MTAs for 6 hours or Oligomycin (1 μM) for 1 hour using a Seahorse Flux Analyzer. Bars represent mean ± SD. (*n*=3) ^*^P<0.05 and ^**^P<0.01 indicate statistical significance.

Mitochondrial dysfunction contributes to the induction of mitophagy [38]. To determine if mitochondrial damage was an upstream initiator of enhanced autophagic flux, MDA-MB-231 and MCF-12A cells were treated with different MTAs to evaluate changes in mitochondrial homeostasis prior to increased autophagy flux (Figure 3). Initial analyses of ΔΨ_m_ revealed that JC-1 aggregate formation was elevated in MDA-MB-231 cells as compared to MCF-12A, which supports the previous observation that mitochondria are hyperpolarized in MDA-MB-231 cancer cells [10]. Moreover, JC-1 fluorometric analysis at six hours demonstrated a selective decline in aggregate formation in MDA-MB-231 cells as compared to MCF-12A (Figure 3A and Supplementary Figure 3a). To verify mitochondrial depolarization, tetramethylrhodamine dimethyl ester (TMRM), a potentiometric fluorescent cation, was quantified and imaged in MDA-MB-231 cells using fluorometrics and confocal microscopy, respectively (Figure 3B and 3C). TMRM accumulates within mitochondria in a membrane potential dependent manner. Alterations to the ΔΨ_m_ can be measured by quantifying the TMRM fluorescence [39, 40] as demonstrated by the loss of TMRM fluorescence intensity in MDA-MB-231 cells treated with CCCP, a known mitochondrial depolarizing agent [41] (Figure 3B). In contrast to TPP treatment, each MTA induced a loss in TMRM fluorescence intensity at as early as three hours in MDA-MB-231 cells (Figure 3B and 3C). These data suggest that MitoQ, MitoCA, and MitoApo cause a loss in ΔΨ_m_ prior to the observed increase in autophagy flux selectivity in MDA-MB-231 cells compared to MCF-12A cells.

To further clarify changes in mitochondrial homeostasis induced by MTAs, mitochondrial protein turnover, morphology, ROS production and oxygen consumption were determined in both cell lines. The effects of MTA treatment on mitochondrial protein turnover and morphology were evaluated in MDA-MB-231 and MCF-12A cells using MitoTracker Red (Supplementary Figure 3b). MitoTracker Red fluorescence did not change in MDA-MB-231 or MCF-12A cells treated with MTAs suggesting that the mitochondria content was comparable to the control at six hours. However, MTA induced changes in the mitochondrial morphology in both cell lines (Figure 3C) in a manner that appears to be similar to previous findings that proposed MTAs induced mitochondrial fission [42]. These data suggest that acute MTA exposure (<6 hours) does not affect mitochondrial turnover but alters the mitochondrial morphology in both MDA-MB-231 and MCF-12A cell lines.

To establish the effects of MTAs on mitochondrial function, the rate of ROS generation and oxygen consumption were measured in MTA treated MDA-MB-231 and MCF-12A cells. MitoSOX, a mitochondrial superoxide indicator, and MitoTracker Green were used to identify changes in the rate of mitochondrial ROS production (Figure 3D and 3E). At six hours, MitoSOX red fluorescence increased in MDA-MB-231 cells treated with each MTA as compared to the control (Figure 3D). However, MitoSOX and MitoTracker co-localization in MDA-MB-231 cells was heterogeneous with cells containing MitoSOX distributed in the cytosol and within the matrix of the mitochondria (Figure 3D and Supplementary Figure 3d). To determine if MTAs induced ROS production was selective for MDA-MB-231 cells as compared to MCF-12A cells, MitoSOX fluorescence was quantified in MDA-MB-231 and MCF-12A cells treated with different MTAs (Figure 3E). Antimycin A, a complex III inhibitor, induces mitochondrial superoxide production as indicated by the increase in the MitoSOX fluorescent intensity in both cell types. ROS generation was elevated in MDA-MB-231 and MCF-12A cells suggesting that MTAs did affect mitochondria ROS production in both cell lines. To assess mitochondrial oxygen consumption, Oligomycin, an F_1_F_0_-ATPase inhibitor, was used as a positive control to block mitochondrial respiration in both cell lines [43] (Figure 3F and Supplementary Figure 3e). Similar to MitoSOX data, both cell lines decreased the rate of oxygen consumption, but only the MDA-MB-231 cells had oxygen consumption rates comparable to Oligomycin. Collectively, these data suggest that MTAs induce mitochondrial dysfunction in both cell lines; however, MCF-12A cells were partly resistant to mitochondrial damage and depolarization.

### MTAs induce a loss in mitochondrial alkalinity

**Figure 4:**
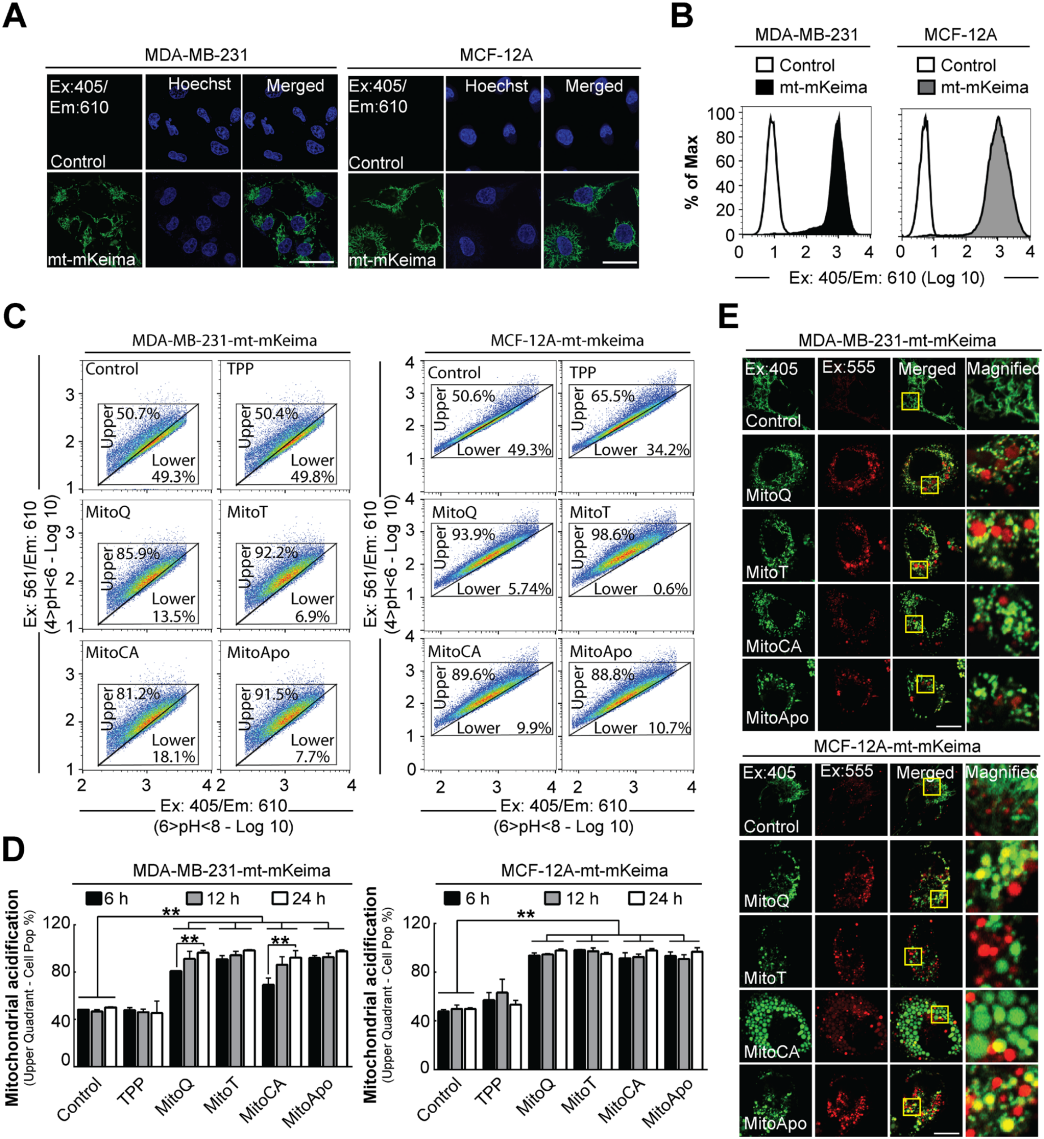
Mitochondrial acidification in stably-expressing mt-mKeima MDA-MB-231 and MCF-12A cells Stable mt-mKeima expressing MDA-MB-231 and MCF-12A cell lines were generated to analyze mitochondrial pH changes in breast cancer and non-cancer cell lines. To verify mitochondrial location, confocal images were captured of stably expressing empty vector (pLVX) or mt-mKeima (pLV-mt-mKeima) **(A)** MDA-MB-231 and MCF-12A cells (Ex: 405 nm/Em: 610 nm). Scale bar is 40 μm. **(B)** Representative FACS histogram of MDA-MB-231 and MCF-12A cells expressing an empty vector or mt-mKeima using Ex:405 nm/Em:610 nm. **(C)** Representative 610 nm emission population analysis of the mt-mKeima pH excitation shift from 405 nm (4>pH<6) to 561 nm (6>pH<8) of the stable cells treated with DMSO (control) or 1 μM MTAs for 12 hours. **(D)** Quantification of mt-mKeima fluorescent shift to the upper quadrant in cell populations at 6, 12 and 24 hours of 1 μM MTA treatments. Bars represent mean ± SD. The number of cells counted for each FACS analysis was 50,000 events per experiment. (*n*=3) **(E)** Representative confocal images (Ex. 405 nm/Em: 610 nm and Ex: 555/Em: 610) of MTA treated cells stably expressing mt-mKeima at 24 hours post treatment. Scale bar is 20 μm. Yellow box indicates the area that was magnified. ^*^P<0.05 and ^**^P<0.01 indicate statistical significance.

A loss in mitochondrial alkalinity precedes mitochondrial autophagy [44]. To investigate if MTA induced a change in the mitochondrial pH, stably expressing mitochondrial targeted mKeima (mt-mKeima) MDA-MB-231 and MCF-12A cell lines were generated and evaluated using a fluorescence-activated cell sorting (FACS) based assay [25, 45, 46]. mKeima, a coral-derived protein, is a pH dependent dual excitation fluorophore with a fixed emission that is resistant to acidic lysosomal proteases [47]. mKeima expression efficiency and localization to the mitochondria in MDA-MB-231 and MCF-12A were confirmed using FACS analysis and confocal microscopy (Figure 4A and 4B). The mKeima pH dependent excitation and emission spectra indicates that mt-mKeima emission under alkaline or neutral conditions (6>pH<8) is more specific for an excitation at 405 nm, in contrast to acidic (4>pH<6) excitation at 561 nm [47]. Under basal conditions, the mt-mKeima fluorescence emissions at both excitations were established for both cell lines and equally subdivided into an upper and lower quadrant. A shift in the cell population fluorescence to the upper quadrant (i.e. a higher Ex:561/Ex405 emission ratio) would indicate a loss in mitochondrial matrix alkalinity as demonstrated in both cell types treated with CCCP, a positive control agent known to induce mitochondrial acidification [48–50] (Supplementary Figure 4a and 4b). The quantification for mt-mKeima FACS analysis is listed Supplementary Tables 1 and 2.

Utilizing the pH specific excitation, stably expressing mt-mKeima MDA-MB-231 and MCF-12A cell populations were analyzed for MTA-induced changes in mitochondrial pH at 6, 12 and 24 hours (Figure 4C and 4D). At six hours post MTA treatment, the mitochondrial alkalinity decreased in both cell types as indicated by the entire cell population relocating to the upper quadrant (Ex: 561/Ex:405 ratio). Prolonging the treatment time to 12 and 24 hours did not re-establish mitochondrial alkalinity as compared to the vehicle control. Confocal microscopy confirmed MTA induced changes in mitochondrial mt-mkeima fluorescence at 24 hours in both cell lines by the formation of acidic red punctae (Ex: 555 nm/Em: 610 nm) that were colocalized with or without alkaline green punctae (Ex: 405 nm/Em: 610 nm) in MTA treated cells at 24 hours (Figure 4E). Collectively, these data suggest that MTAs induced a non-selective cell type decrease in the mitochondrial matrix alkalinity, which does not appear to translate into the selective downstream activation of autophagy in MDA-MB-231 cells.

### MTAs induce lysosomal-dependent mitochondrial degradation

**Figure 5:**
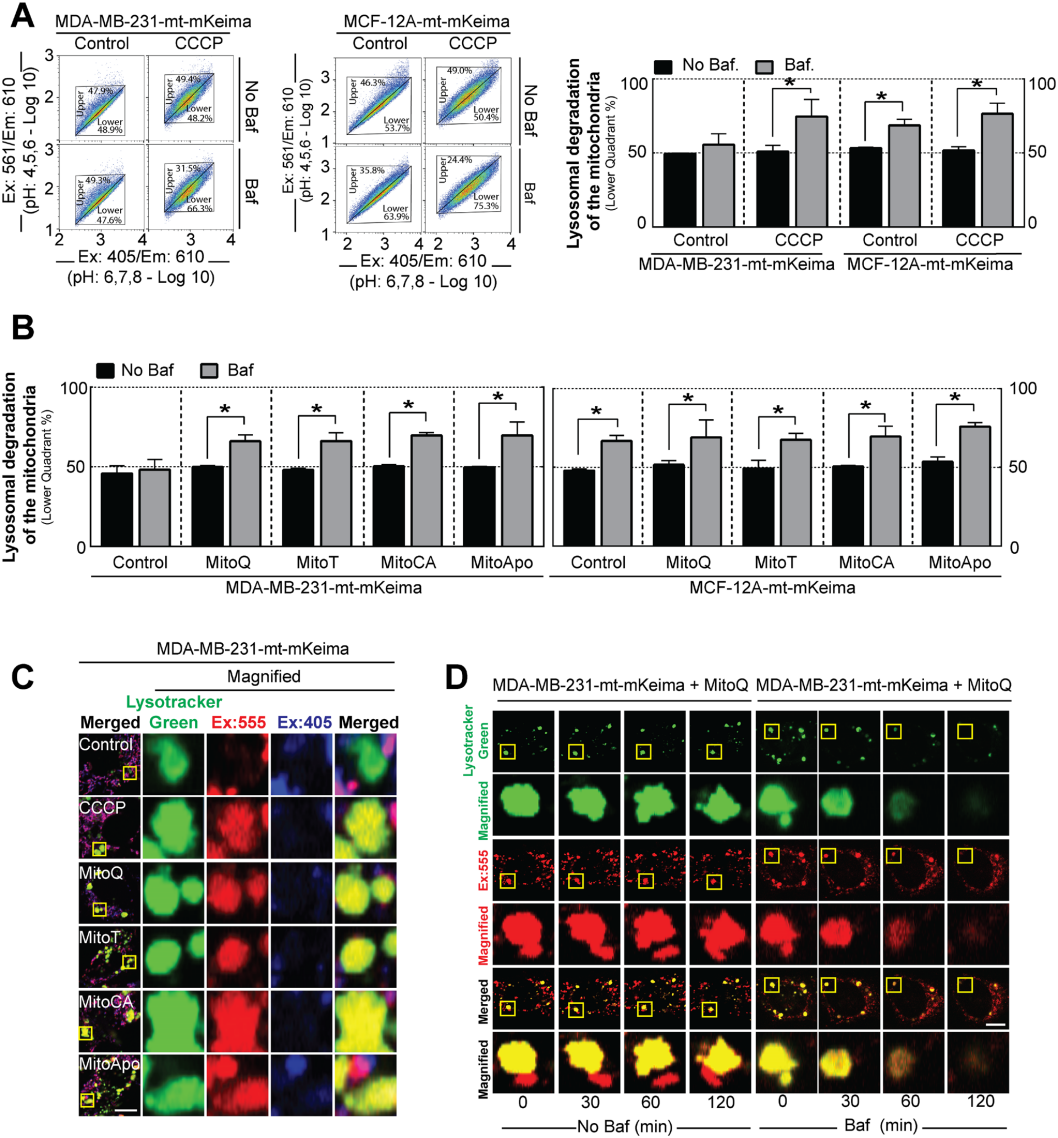
Lysosomal degradation of the mitochondria is selective for MDA-MB-231 cancer cells as compared to MCF-12A healthy cells **(A)** Representative 610 nm emission population analysis of the mt-mKeima pH excitation shift from 405 nm (4>pH<6) to 561 nm (6>pH<8) of the stably expressing MDA-MB-231 and MCF-12A cells treated with or without 30 mM CCCP for 3 hours in the presence or absence of 5 nM Baf for 2 hours prior to analyses. Bar represents the mean ± SD. (*n*=3). **(B)** Cell populations were analyzed for mt-mKeima pH shifts to lower quadrant using MDA-MB-231 and MCF-12A cells treated with 1 μM of MTAs for 12 hours in the presence 5 nM Baf for 2 hours prior to analysis. Bar represents the mean ± SD. (*n*=3) **(C)** Representative confocal images of mt-mKeima fluorescent emission at 610 nm after excitation at 405 and 555 nm in stably expressing MDA-MB-231 cells treated with different MTAs at 1 μM for 24 hours in the presence of 100 nM Lysotracker Green. Scale bar is 10 μm. **(D)** Kinetic confocal analysis of mt-mKeima fluorescent emission at 610 nm after excitation at 555 nm in stably expressing MDA-MB-231 cells treated with MitoQ at 1 μM for 24 hours in the presence of 100 nM Lysotracker with and without Baf. Scale bar is 10 μm. ^*^P<0.05, and ^**^P<0.01 indicate statistical significance.

To investigate if lysosomes are degrading mitochondria, mt-mKeima expressing MDA-MB-231 and MCF-12A cells were treated with MTAs in combination with Baf to neutralize the lysosomes. Neutralization of the acidic lysosomal vacuole by Baf would cause diminution in mKeima emission at Ex:561 and a cell population shift to the lower quadrant (Ex:561/Ex:405 ratio). Under basal conditions, LC3-II accumulation in the presence of Baf indicates both cell lines have basal autophagic flux (Supplementary Figure 5a), but only the MCF-12A cells decreased the mt-mKeima fluorescent emission ratio (Ex:561/Ex:405) indicated by the accumulation of cells located in the lower quadrant, thus suggesting lysosomal dependent mitochondrial degradation (Figure 5A). Serum starvation and rapamycin treatment, two known non-selective autophagy stimulators, in the presence of Baf did not affect the mt-mKeima fluorescence ratio in the MDA-MB-231 cell population (Supplementary Figure 5b). Next, mt-mkeima expressing cells were subjected to CCCP to induce selective mitophagy in the presence of Baf (Figure 5A). Lysosome neutralization caused a decline in the Ex:561/Ex:405 emission ratio and an increase in the percentage of MDA-MB-231 cells located the lower quadrant indicating that CCCP treatment in induced lysosomal dependent mitochondrial degradation. Collectively, these data suggest that under these conditions either MDA-MB-231 cells do not undergo basal mitochondrial degradation or that the rate of mitochondrial degradation is significantly lower in MDA-MB-231 cells as compared to MCF-12A.

To determine if MTAs induced lysosomal dependent mitochondrial degradation, mt-mKeima expressing MDA-MB-231 and MCF-12A cells were subjected to the MTA treatments for 12 hours in the presence of Baf (Supplementary Figure 5c and 5b). In contrast to the vehicle control, MDA-MB-231 cells exposed to MTAs decreased the Ex:561/Ex:405 emission ratio and increased the percentage of cells in the lower quadrant in the presence of Baf. These results indicate that MTA treatment activated or increased mitochondrial degradation via the lysosome in MDA-MB-231 cells. In contrast to MDA-MB-231 cells, MCF-12A cells exposed to the vehicle control and MTAs increased the percentage of cells in the lower quadrant in the presence of Baf indicating the degradation of mitochondria. Confocal microscopy of Lysotracker Green, a lysosomal dye, in MDA-MB-231 cells expressing mt-mKeima verified colocalization of acidic mt-mKeima fluorescence with the lysosomes following MTA exposure (Figure 5C and 5D). The addition of Baf to these cells resulted in a loss of fluorescence signals of mt-mKeima and lysotracker green (Figure 5D and Supplementary Figure 5e). This observation was consistent with the previous FACS analysis data (Figure 5B). Collectively, these data suggest that MTA-induced lysosomal dependent mitochondrial degradation is a response selective to MDA-MB-231 but not to MCF-12A cells.

### MTAs induce mitophagy in MDA-MB-231 cells

**Figure 6:**
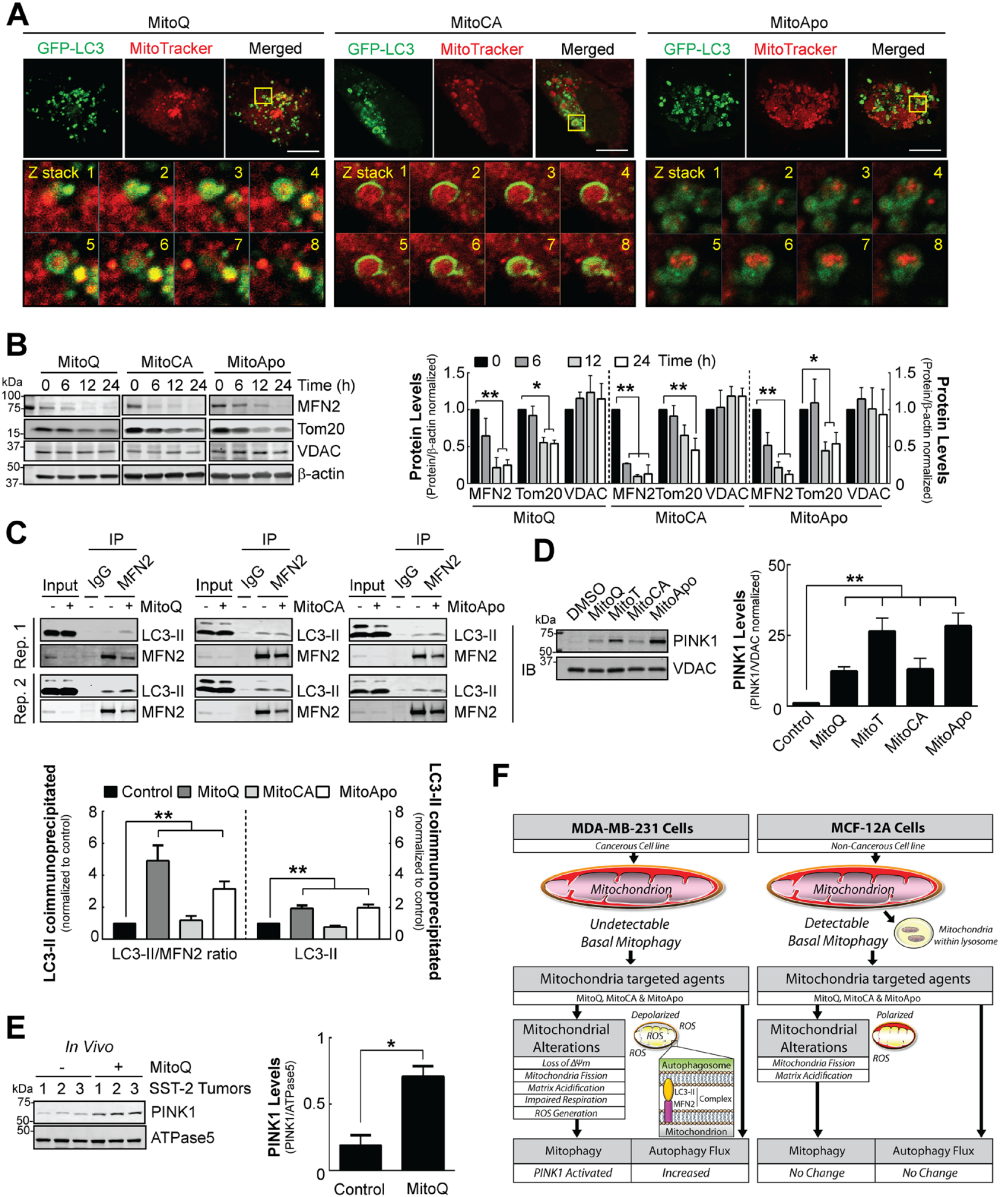
Mitophagy execution via PINK1 accumulation, MFN2/TOM20 reduction and mitophagy signaling in MDA-MB-231 cells *in vitro* and SST2 tumors *in vivo* **(A)** Confocal images of GFP-LC3 expressing MDA-MB-231 cells preloaded with MitoTracker Red at 12 hours post MTA treatment. Scale bar is 10 μm. Yellow box indicates the area selected for obtaining z stacks. **(B)** Cropped immunoblot of the outer mitochondrial membrane proteins MFN2, TOM20 and VDAC in MDA-MB-231 cells treated with 1 μM of different MTAs at the indicated times. Bars represent the mean ± SD SEM. (*n*=3) **(C)** Representative cropped LC3 immunoblots of a mitochondrial extract (300 μg) after MFN2 immunoprecipitation from MDA-MB-231 cells exposed to 1 μM of different MTAs for 24 hours to confirm an endogenous protein complex interaction between the autophagosome and mitochondria. Bar represents the mean ± SEM. Two replicates (Rep.) are shown. (*n*=3) **(D)** Representative cropped PINK1 immunoblot from the mitochondrial fractions of cells treated with different MTAs for 12 hours. Bar represents the mean ± SEM. (*n*=4) **(E)** Representative cropped PINK1 immunoblot from tumor mitochondrial extracts using a rat SST-2 allograft model after DMSO or MitoQ treatment for 14 days. Bar represents the mean ± SD. (*n*=3) ^*^P<0.05, and ^**^P<0.01 indicate statistical significance. **(F)** Schematic model for the proposed mechanism for the mitophagy selective response induced by MTA in MDA-MB-231 cells as compared to MCF-12A cells (See Discussion).

Mitochondria can be delivered to the lysosome via autophagosomes containing LC3 or mitochondrial derived vesicles that lack LC3 [19, 51]. To determine if MTA treatments led to mitophagy, GFP-LC3 expressing MDA-MB-231 cells were preloaded with MitoTracker Red followed by MTA treatment for 12 hours to reveal mitochondria located within autophagosomes (Figure 6A). Outer mitochondrial membrane proteins facilitate a direct linkage between the mitochondria and autophagosomes via a protein complex containing adaptor proteins [25, 52]. To identify the outer mitochondrial membrane proteins degraded at least in part by autophagy, MFN2, TOM20 and VDAC protein levels were quantified as likely targets of proteolytic degradation (Figure 6B). In contrast to VDAC, the protein levels of MFN2 and TOM20 declined at 12 and 24 hours post MTA treatment in MDA-MB-231 cells. MFN2 is phosphorylated and ubiquitinated by the PINK1/Parkin mitophagy mechanism to facilitate mitophagy induction [53, 54]. Formation of protein complex between the mitochondria and autophagosomes would suggest selective mitophagy. Therefore, the mitochondrial fraction from MTA treated MDA-MB-231 cells were used for immunoprecipitation of MFN2 followed by LC3 immunoblotting at 24 hours. The data revealed an increase in the interaction between LC3-II and MFN2 (Figure 6C). Moreover, PINK1 accumulation was confirmed in MTA treated MDA-MB-231 cells (Figure 6D).

To translate these *in vitro* findings to an *in vivo* setting, mitochondrial-enriched extracts from rat SST-2 tumors were prepared from a rat allograft breast cancer model treated with a vehicle control or MitoQ (Figure 6E). Immunoblot analysis of PINK1 revealed that MitoQ treatment increased PINK1 protein levels, suggesting that MitoQ may induce mitochondrial dysfunction and mitophagy in rat tumors *in vivo*. Collectively, these data confirm that MTAs induce mitophagy in MDA-MB-231 breast cancer cells.

## DISCUSSION

Our findings provide the first evidence for how molecules that target or accumulate in the mitochondria can selectively damage mitochondria and signal for mitophagic clearance (Figure 6F). In this report, we optimized novel investigational tools that can be applied for the study of mitophagy and anti-cancer therapeutics designed to cause mitochondrial dysfunction. First, we demonstrated a sub-lethal concentration of MitoQ, MitoCA, and MitoApo, but not MitoT can selectively induce autophagy in malignant breast cancer cells as compared to primary mammary cells. Inhibition of autophagy in combination with MitoQ is cytotoxic to MDA-MB-231 cells [1]. This indicates that mitophagy is a survival mechanism in these malignant cancer cells with mitochondrial damage. Furthermore, we established that different redox moieties conjugated to TPP can affect the autophagic response and mitochondrial membrane potential of non-cancerous cells (Figures 1 and 2). This implicates that the structure and chain length of the conjugated molecule attached to TPP may in part determine the cancer cell selectivity of MTAs, which is currently under further investigation. Second, MDA-MB-231 cells are more sensitive to MTAs as compared to MCF-12A (Figure 3). Our kinetic analyses demonstrate that drug-induced mitochondrial damage is an upstream event that leads to mitophagy activation in MDA-MB-231 cells. These results indicate that the onset of enhanced autophagy and mitochondrial damage is not simultaneous in these cancer cells (Figures 2 and 3). Third, therapeutic drug induced mitochondrial damage can lead to the activation of PINK1-induced mitophagy in cells and a tumor-bearing animal model (Figure 6). We identified three molecules that selectively activate mitophagy in a malignant breast cancer cells as compared to healthy breast cells that could be capitalized for further drug discovery studies. Collectively, this study shows that MTAs can be used both as investigational tools and anti-cancer agents to cause mitochondrial dysfunction and mitophagy in malignant breast cancer cells.

Several anti-cancer drugs target mitochondrial metabolism for the treatment of cancer [55]. To improve the efficiency of these drugs, different experimental strategies are emerging to facilitate the delivery of these drugs to the mitochondria [2, 3, 7, 8, 14–17, 30, 42]. For example, TPP conjugated to metformin increased the potency by 1,000 fold in cancer cells both *in vitro* and *in vivo* [2]. Our study demonstrates that mitophagy is a potential target to further increase the efficacy of these drugs. Mitophagy dysfunction in PINK1 knockout MDA-MB-231 cells has demonstrated to enhance cell death following mitochondria damage [22]. Furthermore, mitochondrial targeted redox agents have been reported to provide beneficial antioxidant effects to non-cancerous cells [56]. This suggests that these agents may provide the ability to specifically target and damage cancer cells.

Mitophagy has been observed in many different cell types and diseases. Assessing the rate of mitochondrial degradation or mitophagic flux remains challenging. A new method to quantify mitophagy is mt-mKeima, a dual excitation mitochondrial pH sensitive fluorophore, which is used to monitor the delivery of the mitochondria to the lysosome [25, 45–47]. However, mitochondrial acidification occurs prior to mitophagy [47, 57]. Thus, the change in the mt-mKeima fluorescence could potentially be independent of lysosome. To distinguish mitochondrial acidification from lysosomal dependent mitochondrial degradation in this assay, Bafilomycin was administered to specifically neutralize the lysosomal compartment. FACS analysis and LC3-II immunoblotting in combination with lysosomal neutralization revealed that MDA-MD-231 cells contain basal autophagic flux without lysosomal dependent mitochondrial degradation, in contrast to MCF-12A cells that contain both (Figure 5). However, MTAs and CCCP induced mitochondrial damage that activated mitophagy in MDA-MB-231 cells. Therefore, one can speculate that deficient basal mitophagy in unstressed malignant cancer cells may contribute to the development of aberrant mitochondrial characteristics, such as a hyperpolarized mitochondrial membrane potential and heightened ROS production, found in some cancers. Impaired mitochondrial turnover is known to facilitate the accumulation of defective mitochondria and enhanced ROS generation [19–21]. Further studies focused on cancer cell transformation and lysosomal-dependent mitochondrial degradation may provide further insight into how mitophagy contributes to changes in cancer cell mitochondria and the role of mitophagy in the metabolic shift from mitochondrial respiration to aerobic glycolysis.

Numerous mechanisms can lead to the initiation of mitophagy, however a characteristic of macromitophagy is that autophagosome selectively recognize mitochondria through complex protein to protein interactions [58]. Mitofusin 2 (MFN2) has been identified as a protein that regulates the recognition of mitochondria and autophagic flux [50, 53, 54, 59, 60]. Here we demonstrate for the first time that autophagosomes are recognizing mitochondria via an endogenous protein complex that contains LC3-II and MFN2 in MDA-MB-231 cells. MTA exposed cells simultaneously accumulate PINK1 and increase the LC3-II interaction with MFN2 (Figure 6). This suggests that PINK1 may be activating Parkin to facilitate this interaction, which is currently under investigation. Contrarily, PINK1 accumulation and mitophagy flux were undetectable under non-stressed conditions but the interaction between LC3-II and MFN2 was observed. This may indicate that autophagosomes recognize damaged mitochondria in MDA-MB-231 cells but the downstream process leading to lysosomal degradation is impaired, which requires further investigation.

Delineation and characterization of mitochondrial dysfunction between normal and diseased cells could facilitate the development of therapeutic strategies targeting mitophagy [21]. MTA treatment revealed characteristics of acute mitochondrial dysfunction in both cancerous and non-cancerous breast cell lines with the exception of ΔΨ_m_. In contrast to MDA-MB-231 cells, MCF-12A cells were actively undergoing mitophagy in the absence of MTA treatment. This may reduce the susceptibility of MCF-12A cells to MTA-induced mitochondrial damage by chronically activated basal levels of mitophagy removing dysfunctional mitochondria. Several potential therapeutics have been studied as mitophagy-activating compounds with distinct mechanisms, but current approaches to inhibit mitophagy appear to be limited to peptide inhibitors that disrupt the recognition of dysfunctional mitochondria or mitochondrial division inhibitors that suppress fission [61]. This unmet need is at least in part due to lack of scientific approaches to assess mitophagy inhibition, thus utilizing the mt-mKeima assay optimized in this paper may facilitate the development of both novel mitophagy activating agents and inhibitory drugs. The development of pharmacological interventions to inhibit mitophagy in combination with chemotherapeutics in cancer cells selectively warrants further studies for specific cancer types. Collectively, this investigation demonstrates that MitoQ, MitoCA and MitoApo-induced mitochondrial dysfunction activated mitophagy in MDA-MB-231 cells, and mitophagy suppression may improve the therapeutic efficacy of these drugs for anti-cancer therapies.

## MATERIALS AND METHODS

### Cell culture

MDA-MB-231 and MCF-12A cells were obtained from ATCC. MDA-MB-231 cells were cultured in DMEM/F-12 (Mediatech) containing L-glutamate, sodium pyruvate, 10% fetal bovine serum and penicillin/streptomycin. MCF-12A cells were maintained in mammary epithelia cell growth media (Lonza) with 10% fetal bovine serum.

### Immunoblotting

To determine autophagy flux, proteins were harvested using radio immunoprecipitation (RIPA) buffer with protease and phosphatase inhibitors. Immunoblotting was performed using β-actin, VDAC, LC3B (Cell Signaling-Cat: 3700, 4866, 2775), PINK1 (Novus Biologicals-Cat: BC100494) MFN2, ATPase5 (AbCam-Cat: ab56889, ab14748) and Tom20 (Santa Cruz-Cat: sc17746). Images were collected using an Odyssey imager (LI-COR). Densitometry was analyzed using Image J software.

### Mitochondrial ROS production

Cells were treated with 2 μM MitoSOX and 5 μM Hoechst (Thermo Fisher) for 20 minutes prior to analysis. Cells were washed twice in PBS followed by the addition of phenol red free media prior to spectroscopy measurements or confocal microscopy imaging as described by the manufacturer.

### Mitochondrial membrane potential, protein turnover and morphology

Cells were treated with JC-1 as previously described [1]. Briefly, MTA treated cells were incubated with JC-1 for 20 minutes followed by two washes using phenol red free cell medium prior to emission measurements. Emission was measured at 590 nM and 540 nM after excitation at 480 nM using a SpectraMax i3 (Molecular Devices). To measure mitochondrial membrane potential 100 nM of TMRM was preloaded into cells for 20 minutes followed by two washes in PBS then cell culture media with DMSO or MTAs. Hoechst stain (Thermo Fisher) was added 20 minutes prior to the emission measurement. Quantification of TMRM and Hoechst fluorescence was measured using Celigo, a cell imager cytometer, (Nexcelom). To measure mitochondrial protein turnover, cells were preloaded for 20 minutes with 30 nM MitoTracker Red (Thermo Fisher) prior to MTA treatments. Hoechst stain was added 20 minutes prior to the completion of the experiment for normalization of fluorescent intensity.

### Mitochondrial oxygen consumption

MDA-MB-231 and MCF12A cells were seeded at 8 x 10^5^ for 12 hours prior to measuring mitochondrial oxygen consumption using a Seahorse XF^96^ analyzer (Agilent). Cells were treated with MTA for 6 hours or Oligomycin for 1 hour prior to measuring oxygen consumption as described by manufacturer.

### GFP-LC3 Transfection and generation of stable mt-mKeima cell lines

GFP-LC3 was transfected into MDA-MB-231 and MCF-12A cells using DharmaFECT Duo (Dharmacon) following manufacturer’s protocol. GFP-LC3 was a generous gift from Dr. William Dunn, JR at the University of Florida (Gainesville, FL). Transfected cells were incubated 24 hours prior to addition of any treatment.

### Live cell confocal microscopy

For confocal imaging experiments, 5 x 10^5^ cells were seeded on fibronectin/gelatin coated glass bottom dishes (Cellvis) for 12 hours prior to any fluorophore or drug treatments. Lysotracker (Thermo Fisher) was added 20 minutes prior imaging. Transfected cells were grown for 24 hours prior to MTA treatment. Images were taken on an inverted Zeiss LSM 700 confocal microscope with an integrated humidified incubation chamber containing 5% CO_2_ at 37°C.

### Mt-mKeima mitophagy assay

The mt-mKeima lentiviral plasmid was a generous gift from Drs. Matsushi at the RIKEN Institute (Wako city, Japan) and Finkel at the National Institute of Health (Bethesda, MD). Stable mt-mKeima MCF-12A and MDA-MB-231 cells were generated using the Lenti-X HTX system (Clonetech) following manufacturer’s protocol. Stably expressing MDA-MB-231 and MCF12A cells treated with different MTA were seeded at 7x10^5^ for FACS analysis using a FlowJo software (v.10.1) and BD FACS Diva software on a BD LSRFORTESSA X-20 (BD Sciences). FACS analysis was performed as described previously with modifications [25]. Upon the completion of the treatments, cells were washed once with PBS and trypsinized. The trypsin was neutralized and cells were pelleted using centrifugation at 0.3 x g for 3 minutes at 4°C. Cells were then washed twice in PBS prior to the addition of sorting buffer (145 mM NaCl, 5 mM KCl, 1.8 mM CaCl2, 0.8 mM MgCl2, 10 mM HEPES, 10 mM Glucose, 0.1% BSA). Dual-excitation ratiometrics of mt-mKeima fluorescence measurements at 405 and 561 nm lasers with 610/20 and 670/30 nm emission filters, respectively. For each sample, 50,000 events were collected and >95% of the cells were gated as a single cell population expressing mt-mKeima fluorescence at excitation 405 nm with an emission 610 nm. Gating for quadrants remained consistent for each experimental condition with approximately 50% of the cell population located in the upper and lower quadrants.

### Mitochondrial extraction and immunoprecipitation

Mitochondria were extracted as described previously [30]. Briefly, cells were trypsinized, washed twice in PBS and incubated with swelling buffer (1 mM Tris pH 7.4) for 20 minutes prior to homogenization. Samples were centrifuged at 1000 x g for 5 minutes twice to collect the supernatant. To collect an enhanced mitochondrial fraction, supernatant were transferred to tubes containing 15 μl of 1.5 M sucrose layer for an additional centrifugation at 14,000 x g for 15 minutes. The supernatant was centrifuged at 21,000 x g for 15 minutes to obtain a cytosolic fraction. The pellet was collected and washed to obtain a mitochondrial pellet, which was lysed in Cell lysis buffer (Cell Signaling). Mouse tumors were collected as previously described [16]. For mitochondrial extraction using rat tumors, the tissue underwent a similar extraction as described for cells with the addition of 2 extra washes in buffer at 1000 x g for 5 minutes post homogenization to remove additional tissue/cell debris.

For mitochondrial extraction followed by immunoprecipitation, 20 million cells were seeded overnight in four different T-600 flasks (EMDMillipore) to achieve approximately 120 million cells per condition. Without any freeze thaw cycles, cells underwent mitochondrial extraction and lysed with cell lysis buffer containing protease and phosphatase inhibitors to obtain 250 μg for immunoprecipitation with 2 μg of MFN-2 or normal mouse IgG antibody (Novus). Samples were incubated and rotated for 16 hours at 4°C followed by the addition of 18 μL of Dynabeads magnetic mouse IgG beads (Thermo Fischer) for an additional 2 hours. Beads were washed 5 times with Cell lysis buffer prior to preparation for immunoblotting.

### Animals

Female SHRs (10 weeks of age) were obtained, housed, and fed as previously described [16]. The investigational protocol was approved by the Institutional Animal Care and Use Committee, Center for Drug Evaluation and Research, FDA, and conducted in an AAALAC accredited facility. All procedures for animal care and housing were in compliance with the Guide for the Care and Use of Laboratory Animals, 1996 (Institute of Laboratory Animal Resources).

### Reagents and statistics

MitoQ, MitoT, MitoCA and MitoApo were kind gifts from Drs. Joseph and Kalyanaraman at the University of Wisconsin Medical College. All other reagents and chemicals that were purchased from Sigma Aldrich. Statistical significance were determined using GraphPad Prism 6 from at least three independently performed experiments. A student’s T test or ANOVA with Tukey’s post-test with a 95% confidence interval was used.

## SUPPLEMENTARY MATERIALS FIGURES AND TABLES







## Abbreviations

MTAs: Mitochondria-targeted agents
Mt-mKeima: Mitochondria-targeted monomeric Keima
TPP: Triphenylphosphonium
ΔΨ_m_: Mitochondrial membrane potential
MitoQ: Mitoquinone
MitoT: Mitotempol
MitoCA: Mitochromanol acetate
MitoApo: Mitoapocynin
ROS: Reactive oxygen species
Baf: Bafilomycin A1
PINK1: Phosphatase and tensin homolog inducible Kinase 1
LC3: Microtubule associated light chain 3
MFN2: Mitofusin 2
VDAC: Voltage dependent anion channel
TOM20: Translocase of the outer membrane 20
GFP: Green fluorescent protein
