# Supplementary material for: Mitochondrial dysfunction activates lysosomal-dependent mitophagy selectively in cancer cells
Source: Oncotarget. 2017 Dec 11;9(1):995–1011. doi: 10.18632/oncotarget.23171 (PMC5787530; doi:10.18632/oncotarget.23171)
Supplement: Supplementary file 1 [file oncotarget-09-995-s001.pdf]

Mitochondrial dysfunction activates lysosomal-dependent mitophagy selectively in cancer cells

SUPPLEMENTARY MATERIALS

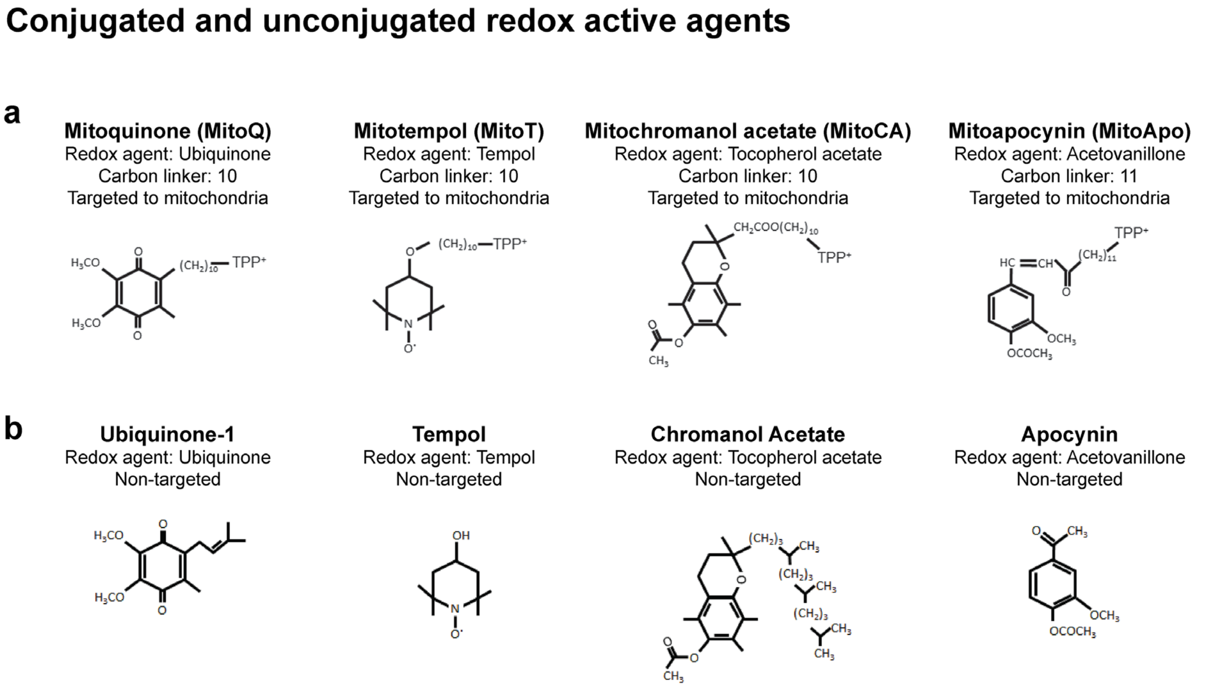

Supplementary Figure 1: Chemical Structures of redox active molecules conjugated and unconjugated to the mitochondrial targeting moiety. (a) The structure of the mitochondria-targeted redox active molecules: mitoquinone, mitotempol, mitochromanol acetate and mitoapocynin. **(b)** The structure of the redox active molecules: ubiquinone, tempol, chromanol acetate, and apocynin unconjugated to a carbon linker chain and triphenylphosphonium.

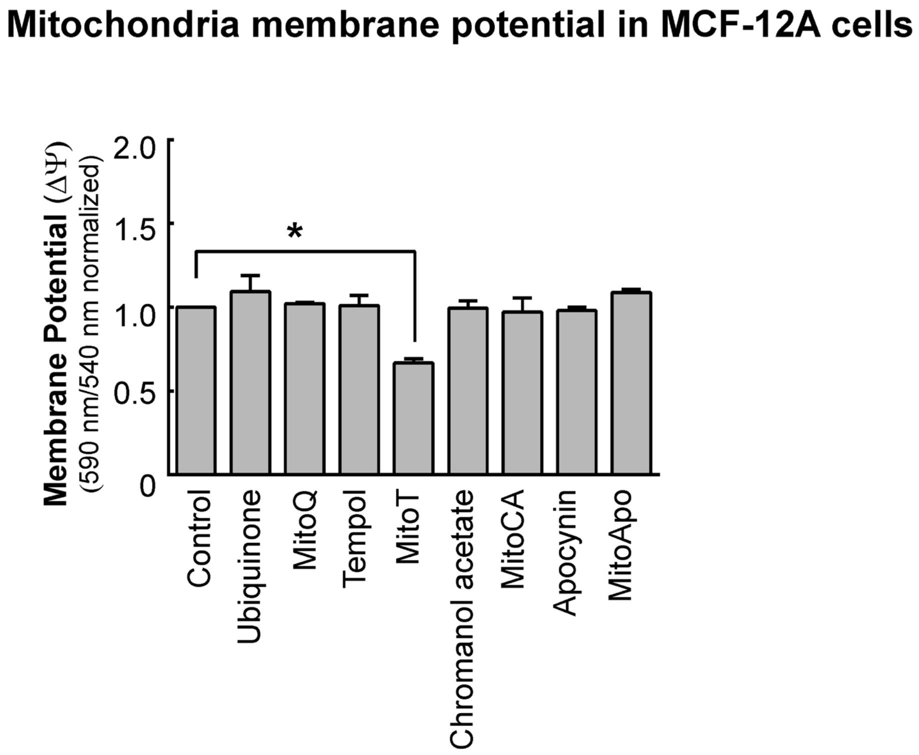

Supplementary Figure 2: Autophagic flux and mitochondrial depolarization in MDA-MB-231 and MCF-12A cells. JC-1 analysis of mitochondrial membrane potential in MCF-12A cells treated with 1 μM of ubiquinone, MitoQ, tempol, MitoT, chromanol acetate, MitoCA, apocynin, and MitoApo for 24 hours. Bar represents mean ± SEM. (*n*=3). $^{*}P<0.05$ indicates statistical significance.

MTA induced mitochondrial dysfunction

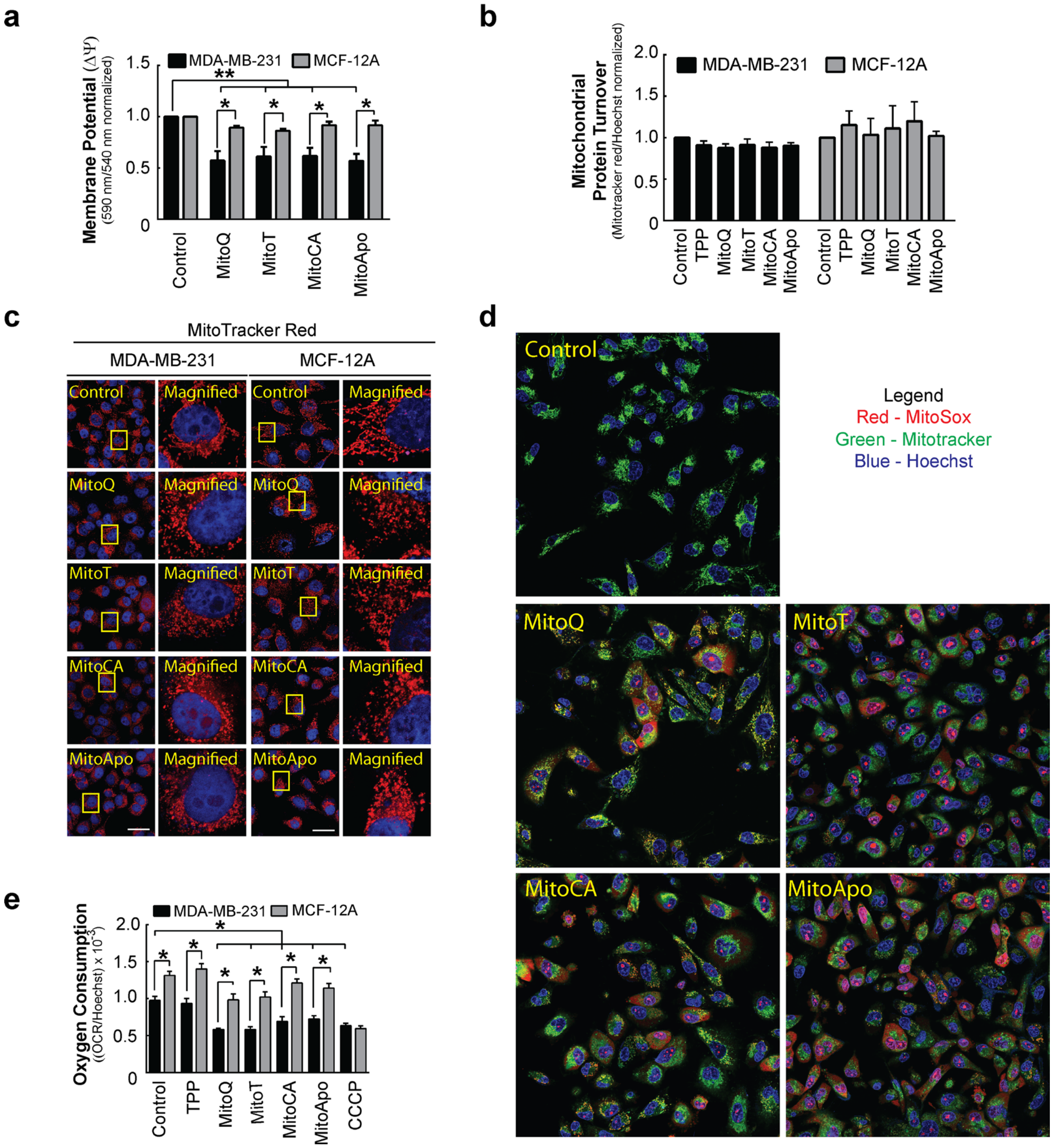

Supplementary Figure 3: MTAs induce a loss in mitochondria function but not mitochondrial content at 6 hours in MDA-MB-231 cells as compared to MCF-12A cells. **(a)** Mitochondria membrane potential was determined using JC-1 fluorometrics in MDA-MB-231 and MCF-12A cells treated with 1 µM of MitoQ, MitoT, MitoCA and MitoApo for 6 hours. Bar represents mean ± SEM. (*n*=3). **(b)** Mitochondria protein turnover was determined by fluorescent emission in MitoTracker red preloaded MDA-MB-231 and MCF-12A cells treated with 1 µM of MitoQ, MitoT, MitoCA and MitoApo for 6 hours Bar represents mean ± SD. (*n*=3). **(c)** Representative confocal images of MitoTracker red preloaded MDA-MB-231 and MCF-12A cells following 6 hours treatment with each MTA at 1 µM. Scale bar is 40 µM. Yellow box is area selected for magnification. **(d)** Large (3x3 tiles stitched) merged confocal images of MTA treated MDA-MB-231cells stained with MitoTracker green, MitoSox and Hoechst. Scale bar is 100 µm. **(e)** Oxygen consumption rate (pmol/min) was measured using the Seahorse XF96 Flux analyzer, while Hoechst fluorescent emission was determined to normalize the mitochondrial function per cell in MDA-MB-231 and MCF-12A cells after 6 hours of 1 µM MTA treatment. Bars represent mean ± SEM (*n*=3). $^{*}P$<0.05 and $^{**}P$<0.01 indicates statistical significance.

CCCP induced mitochondrial acidification

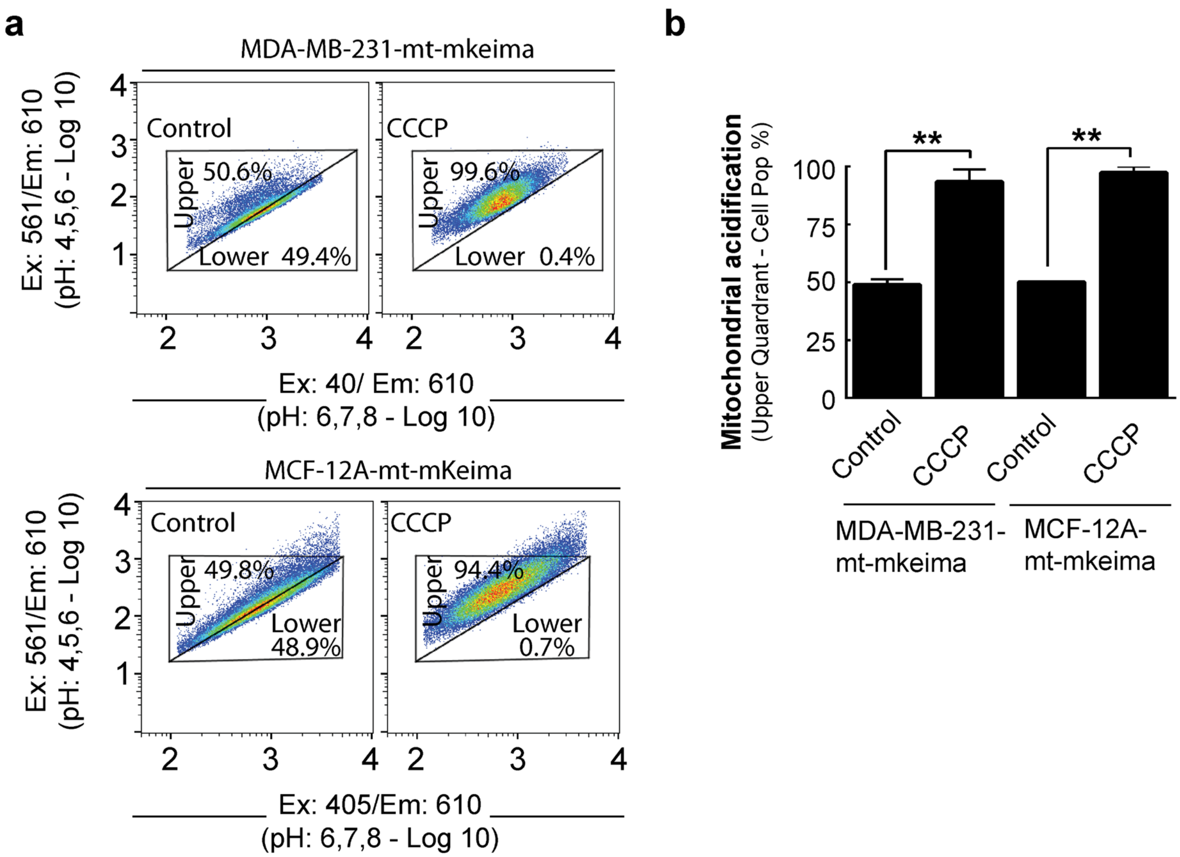

Supplementary Figure 4: Mitochondrial pH changes in stably expressing mt-mKeima MDA-MB-231 and MCF-12A cells treated CCCP. **(a)** Representative cell population dot plots for MDA-MB-231 and MCF-12A cells expressing mt-mKeima treated with 30 µM CCCP for 3 hours. **(b)** Quantification of three individual experiments for the percentage of cells that shifted into the upper (4>pH<6) quadrant. Bar is mean ± SD (*n*=3). $^{**}P$<0.01 indicates statistical significance.

MTA induced lysosomal dependent mitochondrial degradation

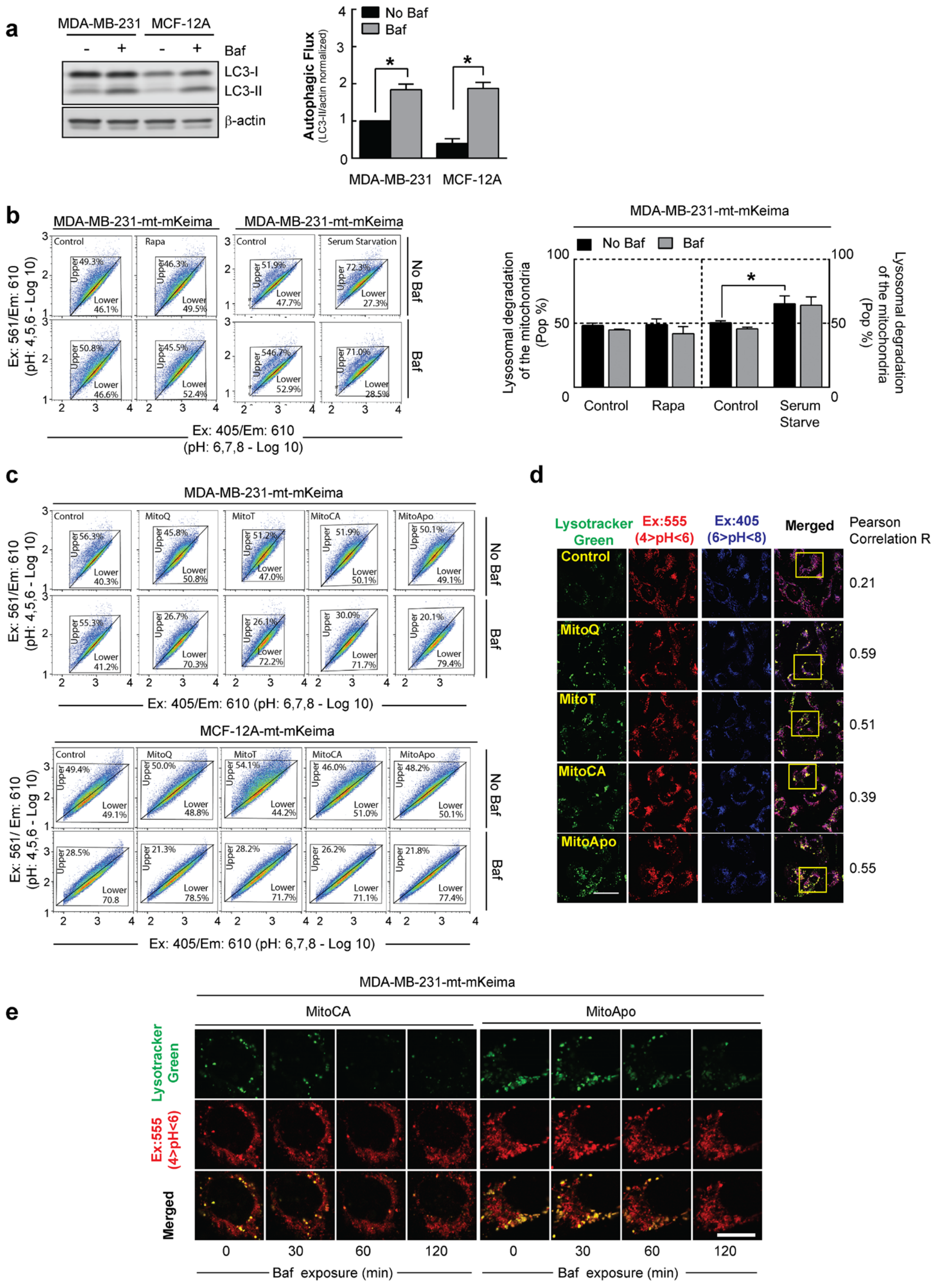

Supplementary Figure 5: MTAs induce mitophagy. (a) Representative cropped LC3 immunoblot of MDA-MB-231 and MCF-12A cells without any MTA treatments in the presence and absence of 5 nM Baf for 2 hours. Bars represent mean ± SD. (n=3). **(b)** Representative MDA-MB-231 cell population analyses in the presence and absence of 100 nM Rapamycin or serum starvation for 24 hours with and without 5 nM Baf. for the final 2 hours. Graph depicts the cell population shift from three independently performed experiments. Bar is the ± and SD (*n*=3). Upper and lower quadrants were set on the non-Baf. treated cells. **(c)** Representative MDA-MB-231 and MCF-12A cell population analyses after 12 hours of DMSO or MTA treatment in the presence or absence of 5 nM Baf. for the final 2 hours. **(d)** Confocal image of mt-mKeima expressing MDA-MB-231 cells treated with the indicated MTAs at 1 µM for 24 hours in the presence of lysotracker green (100 nM). Scale bar is 20 µM. **(e)** Kinetic confocal analysis of MTA treated MDA-MB-231 cells expressing mt-mKeima in the presence of Lysotracker in the presence of Baf. Scale bar is 10 µM. $^{*}P<0.05$ indicates statistical significance.

Supplementary Table 1: MDA-MB-231 stable expressing mt-mKeima FACS analysis

Table describes the percentage of the MDA-MB-231 cell population in the upper and lower quadrants from three independently performed FACs analyses. Cell count was 50,000 events per condition per experiment. **(i-iii)** MDA-MB-231 cells were treated with DMSO (control) or 1 μM of different MTAs for **(i)** 6, **(ii)** 12 and **(iii)** 24 hours prior to FACS analysis. **(iv)** Upper and lower quadrants were set using control cells. Cells were treated with or without 30 μM CCCP for 3 hours. **(v)** MDA-MB-231 cells were treated with or without 30 μM of CCCP for 3 hours in the presence or absence of 5 nM Baf. Non-Baf. treated cells were used to set the upper and lower quadrants. **(vi)** Cells were treated with 1 μM MTA for 10 hours followed by 5 nM Baf. for 2 hours prior to analysis. Non-Baf. treated cells were used to set the upper and lower quadrants. **(vii)** Cells were treated with and without 100 nM rapamycin or **(viii)** serum starved for 24 hours in the presence and absence of 5 nM Baf. for the final 2 hours. Non-Baf. treated cells were used to establish the upper and lower quadrants.

See Supplementary File 1

Supplementary Table 2: MCF-12A stable expressing mt-mKeima FACS analysis

Table describes the percentage of the MCF-12A cell population in the upper and lower quadrants from three independently performed FACS analyses. Cell count was 50,000 events per condition per experiment. **(i-iii)** MCF-12A cells were treated with DMSO (control) or 1 μM of different MTAs for **(i)** 6, **(ii)** 12 and **(iii)** 24 hours prior to FACS analysis. (**iv**) Upper and lower quadrants were set using control cells. Cells were treated with or without 30 μM CCCP for 3 hours. **(v)** MCF-12A cells were treated with or without 30 μM of CCCP for 3 hours in the presence or absence of 5 nM Baf. Non-Baf. treated cells were used to set the upper and lower quadrants. **(vi)** Cells were treated with 1 μM MTA for 10 hours followed by 5 nM Baf. for 2 hours prior to analysis. Non-Baf. treated cells were used to set the upper and lower quadrants.

See Supplementary File 2
