# Supplementary material for: Mitochondrial dysfunction activates lysosomal-dependent mitophagy selectively in cancer cells
Source: Oncotarget. 2017 Dec 11;9(1):995–1011. doi: 10.18632/oncotarget.23171 (PMC5787530; doi:10.18632/oncotarget.23171)
Supplement: Supplementary file 2 [file oncotarget-09-995-s002.doc]

| **Stable Mt-mKeima Expressing MDA-MB-231 FACS Analysis** | | | | | | | | | | | | | |
| --- | --- | --- | --- | --- | --- | --- | --- | --- | --- | --- | --- | --- | --- |
|
| ***i*** | ***MTA treated MDA-MB-231 cells - 6 h*** | | | | | | | | | | | | |
|  |
|  |  | Trail 1 | | | Trial 2 | | Trial 3 | |  | Collective | | | |
|  |  | Quadrant | | | Quadrant | | Quadrant | |  | Upper Quadrant | | Lower Quadrant | |
|  |  | Upper | Lower | | Upper | Lower | Upper | Lower |  | Average | Stan. Dev. | Average | Stan. Dev. |
|  | Control | 48.0% | 48.5% | | 48.5% | 51.8% | 48.4% | 51.8% |  | 48.3% | 0.00 | 50.7% | 0.02 |
|  | TPP | 50.3% | 47.3% | | 46.6% | 53.5% | 46.6% | 53.5% |  | 47.8% | 0.02 | 51.4% | 0.04 |
|  | MitoQ | 80.9% | 17.2% | | 80.8% | 19.2% | 80.8% | 19.2% |  | 80.8% | 0.00 | 18.5% | 0.01 |
|  | MitoT | 87.5% | 11.2% | | 92.7% | 6.5% | 92.7% | 6.6% |  | 91.0% | 0.03 | 8.1% | 0.03 |
|  | MitoCA | 69.3% | 28.5% | | 75.0% | 25.0% | 63.5% | 42.0% |  | 69.3% | 0.06 | 31.8% | 0.09 |
|  | MitoApo | 89.7% | 89.6% | | 93.1% | 6.4% | 93.1% | 6.4% |  | 92.0% | 0.02 | 34.2% | 0.48 |
| ***ii*** | ***MTA treated MDA-MB-231 cells - 12 h*** | | | | | | | | | | | | |
|  |
|  |  | Trail 1 | | | Trial 2 | | Trial 3 | |  | Collective | | | |
|  |  | Quadrant | | | Quadrant | | Quadrant | |  | Upper Quadrant | | Lower Quadrant | |
|  |  | Upper | | Lower | Upper | Lower | Upper | Lower |  | Average | Stan. Dev. | Average | Stan. Dev. |
|  | Control | 45.0% | | 54.8% | 47.4% | 52.2% | 47.9% | 51.8% |  | 46.8% | 0.02 | 52.9% | 0.02 |
|  | TPP | 43.2% | | 56.6% | 47.1% | 52.6% | 48.1% | 51.6% |  | 46.1% | 0.03 | 53.6% | 0.03 |
|  | MitoQ | 93.9% | | 5.6% | 83.9% | 15.7% | 95.7% | 2.8% |  | 91.2% | 0.06 | 8.0% | 0.07 |
|  | MitoT | 97.5% | | 1.4% | 90.8% | 8.5% | 94.6% | 3.6% |  | 94.3% | 0.03 | 4.5% | 0.04 |
|  | MitoCA | 87.0% | | 11.9% | 78.9% | 20.6% | 92.6% | 5.5% |  | 86.2% | 0.07 | 12.7% | 0.08 |
|  | MitoApo | 91.9% | | 1.8% | 90.0% | 9.4% | 96.3% | 0.3% |  | 92.7% | 0.03 | 3.8% | 0.05 |
| ***iii*** | ***MTA treated MDA-MB-231 cells - 24 h*** | | | | | | | | | | | | |
|  |
|  |  | Trail 1 | | | Trial 2 | | Trial 3 | |  | Collective | | | |
|  |  | Quadrant | | | Quadrant | | Quadrant | |  | Upper Quadrant | | Lower Quadrant | |
|  |  | Upper | | Lower | Upper | Lower | Upper | Lower |  | Average | Stan. Dev. | Average | Stan. Dev. |
|  | Control | 49.5% | | 50.2% | 50.5% | 49.4% | 50.5% | 49.4% |  | 50.2% | 0.01 | 49.7% | 0.00 |
|  | TPP | 53.7% | | 46.0% | 48.4% | 51.5% | 34.0% | 66.0% |  | 45.4% | 0.10 | 54.5% | 0.10 |
|  | MitoQ | 98.6% | | 0.7% | 96.1% | 3.6% | 94.3% | 5.6% |  | 96.3% | 0.02 | 3.3% | 0.02 |
|  | MitoT | 98.9% | | 0.1% | 97.7% | 2.0% | 98.3% | 1.5% |  | 98.3% | 0.01 | 1.2% | 0.01 |
|  | MitoCA | 97.4% | | 2.0% | 93.6% | 6.1% | 86.1% | 13.9% |  | 92.4% | 0.06 | 7.3% | 0.06 |
|  | MitoApo | 98.9% | | 0.3% | 96.8% | 2.9% | 97.2% | 2.6% |  | 97.6% | 0.01 | 2.0% | 0.01 |
| ***iv*** | ***CCCP treated MDA-MB-231 cells - 3 h*** | | | | | | | | | | | | |
|  |
|  |  | Trail 1 | |  | Trial 2 |  | Trial 3 |  |  | Collective | | | |
|  |  | Quadrant | | | Quadrant | | Quadrant | |  | Upper Quadrant | | Lower Quadrant | |
|  |  | Upper | | Lower | Upper | Lower | Upper | Lower |  | Average | Stan. Dev. | Average | Stan. Dev. |
|  | Control | 50.60% | | 49.40% | 50.00% | 49.70% | 46.40% | 55.50% |  | 49.0% | 0.02 | 51.5% | 0.03 |
|  | CCCP | 99.60% | | 4.00% | 90.20% | 9.70% | 99.30% | 0.70% |  | 96.4% | 0.05 | 4.8% | 0.05 |
| ***v*** | ***CCCP and Baf. treated MDA-MB-231 cells - 3 h*** | | | | | | | | | | | | |
|  |
|  |  | Trail 1 | | | Trial 2 | | Trial 3 | |  | Collective | | | |
|  |  | Quadrant | | | Quadrant | | Quadrant | |  | Upper Quadrant | | Lower Quadrant | |
|  |  | Upper | | Lower | Upper | Lower | Upper | Lower |  | Average | Stan. Dev. | Average | Stan. Dev. |
|  | Control | 49.0% | | 49.7% | 49.9% | 49.6% | 47.9% | 48.9% |  | 48.9% | 0.01 | 49.4% | 0.00 |
|  | Control + Baf | 36.8% | | 62.1% | 42.0% | 57.2% | 49.3% | 47.6% |  | 42.7% | 0.06 | 55.6% | 0.07 |
|  | CCCP | 42.6% | | 55.8% | 50.3% | 48.9% | 49.4% | 48.2% |  | 47.4% | 0.04 | 51.0% | 0.04 |
|  | CCCP+Baf. | 29.1% | | 69.6% | 10.3% | 87.9% | 31.5% | 66.3% |  | 23.6% | 0.12 | 74.6% | 0.12 |
| ***vi*** | ***MTA and Baf. treated MDA-MB-231 cells - 12 h*** | | | | | | | | | | | | |
|  |
|  |  | Trail 1 | | | Trial 2 | | Trial 3 | |  | Collective | | | |
|  |  | Quadrant | | | Quadrant | | Quadrant | |  | Upper Quadrant | | Lower Quadrant | |
|  |  | Upper | | Lower | Upper | Lower | Upper | Lower |  | Average | Stan. Dev. | Average | Stan. Dev. |
|  | Control | 56.30% | | 40.30% | 48.50% | 47.80% | 50.20% | 49.20% |  | 51.7% | 0.04 | 45.8% | 0.05 |
|  | Control + Baf | 55.30% | | 41.20% | 47.00% | 49.70% | 45.50% | 53.70% |  | 49.3% | 0.05 | 48.2% | 0.06 |
|  | MitoQ | 45.80% | | 50.80% | 47.70% | 51.70% | 50.20% | 49.10% |  | 47.9% | 0.02 | 50.5% | 0.01 |
|  | MitoQ + Baf | 26.70% | | 70.30% | 35.90% | 65.50% | 36.80% | 62.40% |  | 33.1% | 0.06 | 66.1% | 0.04 |
|  | MitoT | 51.20% | | 47.00% | 47.30% | 49.00% | 51.10% | 48.10% |  | 49.9% | 0.02 | 48.0% | 0.01 |
|  | MitoT +Baf | 26.10% | | 72.20% | 33.60% | 63.20% | 36.20% | 63.20% |  | 32.0% | 0.05 | 66.2% | 0.05 |
|  | MitoCA | 51.90% | | 50.10% | 48.80% | 49.20% | 49.30% | 51.50% |  | 50.0% | 0.02 | 50.3% | 0.01 |
|  | MitoCA + Baf | 30.00% | | 71.70% | 28.50% | 69.70% | 32.40% | 68.00% |  | 30.3% | 0.02 | 69.8% | 0.02 |
|  | MitoApo | 50.10% | | 49.10% | 48.90% | 49.80% | 49.80% | 50.10% |  | 49.6% | 0.01 | 49.7% | 0.01 |
|  | MitoAPo + Baf | 20.10% | | 79.40% | 43.40% | 65.30% | 34.80% | 64.70% |  | 32.8% | 0.12 | 69.8% | 0.08 |
| ***vii*** | ***Rapamycin (Rapa) and Baf. treated MDA-MB-231 cells - 24 h*** | | | | | | | | | | | | |
|  |
|  |  | Trail 1 | | | Trial 2 | | Trial 3 | |  | Collective | | | |
|  |  | Quadrant | | | Quadrant | | Quadrant | |  | Upper Quadrant | | Lower Quadrant | |
|  |  | Upper | | Lower | Upper | Lower | Upper | Lower |  | Average | Stan. Dev. | Average | Stan. Dev. |
|  | Control | 45.9% | | 53.6% | 49.3% | 52.2% | 49.3% | 46.1% |  | 48.2% | 0.02 | 50.6% | 0.04 |
|  | Control + Baf | 44.4% | | 55.0% | 47.5% | 53.4% | 50.8% | 46.6% |  | 47.6% | 0.03 | 51.7% | 0.04 |
|  | Rapa | 54.1% | | 45.5% | 46.6% | 50.3% | 46.3% | 49.5% |  | 49.0% | 0.04 | 48.4% | 0.03 |
|  | Rapa + Baf | 44.5% | | 54.8% | 35.6% | 63.6% | 45.5% | 54.4% |  | 41.9% | 0.05 | 57.6% | 0.05 |
| ***viii*** | ***Serum Starvation and Baf. treated MDA-MB-231 cells - 24 h*** | | | | | | | | | | | | |
|  |
|  |  | Trail 1 | | | Trial 2 | | Trial 3 | |  | Collective | | | |
|  |  | Quadrant | | | Quadrant | | Quadrant | |  | Upper Quadrant | | Lower Quadrant | |
|  |  | Upper | | Lower | Upper | Lower | Upper | Lower |  | Average | Stan. Dev. | Average | Stan. Dev. |
|  | Control | 51.9% | | 47.7% | 50.2% | 47.0% | 49.2% | 48.6% |  | 50.4% | 0.01 | 47.8% | 0.01 |
|  | Control + Baf | 46.7% | | 52.9% | 44.2% | 53.1% | 45.6% | 52.4% |  | 45.5% | 0.01 | 52.8% | 0.00 |
|  | Serum Starv. | 72.3% | | 27.3% | 62.5% | 35.0% | 60.9% | 37.2% |  | 65.2% | 0.06 | 33.2% | 0.05 |
|  | Serum Starv. + Baf | 71.0% | | 28.5% | 63.4% | 34.2% | 57.6% | 40.3% |  | 64.0% | 0.07 | 34.3% | 0.06 |
