# Supplementary material for: Mitochondrial dysfunction activates lysosomal-dependent mitophagy selectively in cancer cells
Source: Oncotarget. 2017 Dec 11;9(1):995–1011. doi: 10.18632/oncotarget.23171 (PMC5787530; doi:10.18632/oncotarget.23171)
Supplement: Supplementary file 3 [file oncotarget-09-995-s003.doc]

| **Stable Mt-mKeima Expressing MCF-12A FACS Analysis** | | | | | | | | | | | | |
| --- | --- | --- | --- | --- | --- | --- | --- | --- | --- | --- | --- | --- |
|
| ***i*** | ***MTA treated MCF-12A - 6 h*** | | | | | | | | | | | |
|  |
|  |  | Trail 1 | | Trial 2 | | Trial 3 | |  | Collective | | | |
|  |  | Quadrant | | Quadrant | | Quadrant | |  | Upper Quadrant | | Lower Quadrant | |
|  |  | Upper | Lower | Upper | Lower | Upper | Lower |  | Average | Stan. Dev. | Average | Stan. Dev. |
|  | Control | 49.6% | 51.1% | 46.7% | 52.9% | 47.0% | 51.5% |  | 47.8% | 0.02 | 51.8% | 0.01 |
|  | TPP | 50.1% | 20.4% | 62.1% | 37.3% | 58.9% | 39.3% |  | 57.0% | 0.06 | 32.3% | 0.10 |
|  | MitoQ | 96.1% | 3.7% | 92.8% | 6.6% | 92.1% | 6.8% |  | 93.7% | 0.02 | 5.7% | 0.02 |
|  | MitoT | 98.4% | 0.1% | 97.9% | 0.8% | 98.6% | 0.9% |  | 98.3% | 0.00 | 0.6% | 0.00 |
|  | MitoCA | 96.3% | 3.0% | 90.7% | 8.6% | 87.4% | 11.2% |  | 91.5% | 0.04 | 7.6% | 0.04 |
|  | MitoApo | 96.9% | 3.0% | 92.2% | 7.2% | 90.7% | 8.2% |  | 93.3% | 0.03 | 6.1% | 0.03 |
| ***ii*** | ***MTA treated MCF-12A - 12 h*** | | | | | | | | | | | |
|  |
|  |  | Trail 1 | | Trial 2 | | Trial 3 | |  | Collective | | | |
|  |  | Quadrant | | Quadrant | | Quadrant | |  | Upper Quadrant | | Lower Quadrant | |
|  |  | Upper | Lower | Upper | Lower | Upper | Lower |  | Average | Stan. Dev. | Average | Stan. Dev. |
|  | Control | 52.5% | 48.9% | 46.5% | 52.8% | 50.6% | 49.3% |  | 49.9% | 0.03 | 50.3% | 0.02 |
|  | TPP | 51.1% | 50.1% | 72.9% | 25.5% | 65.5% | 34.2% |  | 63.2% | 0.11 | 36.6% | 0.12 |
|  | MitoQ | 94.5% | 2.0% | 95.2% | 2.8% | 93.9% | 5.7% |  | 94.5% | 0.01 | 3.5% | 0.02 |
|  | MitoT | 99.0% | 0.0% | 94.2% | 0.2% | 98.6% | 0.7% |  | 97.3% | 0.03 | 0.3% | 0.00 |
|  | MitoCA | 93.6% | 2.6% | 94.1% | 3.6% | 89.6% | 9.9% |  | 92.4% | 0.02 | 5.4% | 0.04 |
|  | MitoApo | 89.5% | 8.7% | 94.7% | 3.8% | 88.2% | 10.7% |  | 90.8% | 0.03 | 7.7% | 0.04 |
| ***iii*** | ***MTA treated MCF-12A - 24 h*** | | | | | | | | | | | |
|  |
|  |  | Trail 1 | | Trial 2 | | Trial 3 | |  | Collective | | | |
|  |  | Quadrant | | Quadrant | | Quadrant | |  | Upper Quadrant | | Lower Quadrant | |
|  |  | Upper | Lower | Upper | Lower | Upper | Lower |  | Average | Stan. Dev. | Average | Stan. Dev. |
|  | Control | 48.9% | 50.8% | 49.9% | 49.7% | 50.7% | 47.9% |  | 49.8% | 0.01 | 49.5% | 0.01 |
|  | TPP | 50.8% | 48.9% | 52.0% | 47.7% | 57.3% | 41.3% |  | 53.4% | 0.03 | 46.0% | 0.04 |
|  | MitoQ | 98.7% | 0.3% | 98.3% | 1.1% | 96.4% | 1.4% |  | 97.8% | 0.01 | 0.9% | 0.01 |
|  | MitoT | 96.0% | 0.0% | 95.2% | 0.0% | 93.5% | 0.0% |  | 94.9% | 0.01 | 0.0% | 0.00 |
|  | MitoCA | 98.7% | 0.0% | 98.5% | 0.0% | 96.1% | 0.0% |  | 97.8% | 0.01 | 0.0% | 0.00 |
|  | MitoApo | 98.6% | 0.5% | 98.7% | 0.3% | 92.9% | 5.4% |  | 96.7% | 0.03 | 2.1% | 0.03 |
| ***iv*** | ***CCCP treated MCF-12A - 3 h*** | | | | | | | | | | | |
|  |
|  |  | Trail 1 |  | Trial 2 |  | Trial 3 |  |  | Collective | | | |
|  |  | Quadrant | | Quadrant | | Quadrant | |  | Upper Quadrant | | Lower Quadrant | |
|  |  | Upper | Lower | Upper | Lower | Upper | Lower |  | Average | Stan. Dev. | Average | Stan. Dev. |
|  | Control | 49.80% | 48.90% | 50.10% | 54.20% | 50.40% | 51.60% |  | 50.1% | 0.00 | 51.6% | 0.03 |
|  | CCCP | 94.40% | 0.67% | 98.30% | 0.56% | 90.80% | 9.53% |  | 94.5% | 0.04 | 3.6% | 0.05 |
| ***v*** | ***CCCP and Baf. treated MCF-12A - 3 h*** | | | | | | | | | | | |
|  |
|  |  | Trail 1 | | Trial 2 | | Trial 3 | |  | Collective | | | |
|  |  | Quadrant | | Quadrant | | Quadrant | |  | Upper Quadrant | | Lower Quadrant | |
|  |  | Upper | Lower | Upper | Lower | Upper | Lower |  | Average | Stan. Dev. | Average | Stan. Dev. |
|  | Control | 45.10% | 52.50% | 46.20% | 53.60% | 46.30% | 53.70% |  | 45.9% | 0.01 | 53.3% | 0.01 |
|  | Control + Baf | 27.00% | 71.30% | 30.00% | 70.70% | 35.80% | 63.90% |  | 30.9% | 0.04 | 68.6% | 0.04 |
|  | CCCP | 49.70% | 50.20% | 43.70% | 54.60% | 49.00% | 50.40% |  | 47.5% | 0.03 | 51.7% | 0.02 |
|  | CCCP+Baf. | 5.10% | 94.10% | 28.30% | 70.00% | 24.40% | 75.30% |  | 19.3% | 0.12 | 79.8% | 0.13 |
| ***vi*** | ***MTA and Baf. treated MCF-12A - 12 hrs*** | | | | | | | | | | | |
|  |
|  |  | Trail 1 | | Trial 2 | | Trial 3 | |  | Collective | | | |
|  |  | Quadrant | | Quadrant | | Quadrant | |  | Upper Quadrant | | Lower Quadrant | |
|  |  | Upper | Lower | Upper | Lower | Upper | Lower |  | Average | Stan. Dev. | Average | Stan. Dev. |
|  | Control | 50.20% | 47.30% | 51.00% | 47.50% | 49.40% | 49.10% |  | 50.2% | 0.01 | 48.0% | 0.01 |
|  | Bafilomycin | 34.30% | 64.20% | 44.60% | 55.30% | 28.50% | 70.80% |  | 35.8% | 0.08 | 63.4% | 0.08 |
|  | MitoQ | 46.40% | 53.80% | 46.80% | 52.30% | 50.00% | 48.80% |  | 47.7% | 0.02 | 51.6% | 0.03 |
|  | MitoQ + Baf | 30.90% | 71.80% | 43.00% | 56.70% | 21.30% | 78.50% |  | 31.7% | 0.11 | 69.0% | 0.11 |
|  | MitoT | 44.10% | 54.60% | 46.30% | 49.10% | 54.10% | 44.20% |  | 48.2% | 0.05 | 49.3% | 0.05 |
|  | MitoT +Baf | 32.40% | 67.40% | 3.25% | 96.60% | 28.20% | 71.70% |  | 21.3% | 0.16 | 78.6% | 0.16 |
|  | MitoCA | 50.10% | 50.90% | 46.40% | 49.60% | 46.00% | 51.00% |  | 47.5% | 0.02 | 50.5% | 0.01 |
|  | MitoCA + Baf | 48.10% | 54.40% | 10.70% | 87.20% | 26.30% | 87.10% |  | 28.4% | 0.19 | 76.2% | 0.19 |
|  | MitoApo | 44.80% | 54.60% | 43.30% | 55.90% | 48.20% | 50.10% |  | 45.4% | 0.03 | 53.5% | 0.03 |
|  | MitoAPo + Baf | 27.70% | 72.80% | 12.50% | 87.30% | 21.80% | 77.40% |  | 20.7% | 0.08 | 79.2% | 0.07 |
